# Impact of Biofilm Decontamination Methods on Implant‐Abutment Surface Integrity: A Systematic Review of Quantitative Studies

**DOI:** 10.1111/clr.70077

**Published:** 2025-12-15

**Authors:** Leoluca Valeriani, Arianna Giovannini, Nicola Marco Sforza, Luca Landi, Cristiano Tomasi, Marco Montevecchi

**Affiliations:** ^1^ Department of Biomedical and Neuromotor Sciences, School of Dentistry Alma Mater Studiorum – University of Bologna Bologna Italy; ^2^ Private Practice Bologna Italy; ^3^ Private Practice Rome Italy; ^4^ Department of Periodontology University of Gothenburg, Institute of Odontology Gothenburg Sweden

**Keywords:** dental implants, implant maintenance, supportive peri‐implant care, surface roughness

## Abstract

**Background and Aim:**

To systematically analyze the impact of biofilm disruption and decontamination methods on the surface of dental implants and abutments.

**Methods:**

A systematic search of PubMed, Scopus, and Web of Science was conducted in September 2024. Only quantitative studies analyzing surface alterations caused by physical, mechanical, and chemical decontamination procedures were included. The risk of bias was assessed.

**Results:**

Twenty‐nine studies, all in vitro, were selected. Surface alterations were assessed using profilometer and microscope and were reported through Ra, Rz, and Sa roughness parameters. Differences in surface roughness between control and test groups were evaluated. For titanium machined surfaces, the surface roughness increases or decreases in 96% of cases for Ra and 100% for Sa and Rz, with no consistent pattern. Titanium modified surfaces generally show a reduction trend in roughness parameters following decontamination, though the extent and consistency of this reduction vary across studies. Few studies on zirconia reported minimal surface alteration, with Ra changes between −0.05 and 0.06 and Sa between 0.2 and 0.8 μm. Overall, due to methodological variability, no universally safe tool for maintaining surface integrity could be clearly identified.

**Conclusions:**

The inconclusive and heterogeneous nature of the findings suggests that surface alterations should be considered alongside other factors, rather than as a stand‐alone determinant, when selecting implant surface decontamination methods. Clinical trials are suggested to assess the impact of these alterations on clinical outcomes, such as bacterial growth and biological complications.

## 1 Introduction

Dental implants represent the treatment of choice for managing partial or total edentulism due to their high success and survival rates. However, the success rate of implants could be compromised by the onset of peri‐implant diseases (Renvert et al. 2008), which include peri‐implant mucositis and peri‐implantitis. Peri‐implant mucositis is a biofilm‐induced inflammatory condition of the soft tissue without peri‐implant bone loss, and it is considered a precursor to peri‐implantitis (Jepsen et al. 2015), a condition characterized by both soft tissue inflammation and progressive loss of supporting peri‐implant bone (Berglundh et al. 2018).

These inflammatory conditions, primarily driven by bacterial etiology, exhibit a significant prevalence, with rates reported as 43% for peri‐implant mucositis and 22% for peri‐implantitis (Derks and Tomasi 2015). Given the potential progression from peri‐implant mucositis to peri‐implantitis, effective biofilm removal and maintenance protocols are essential for preserving peri‐implant health and preventing disease progression. Supportive peri‐implant care plays a central role in this context, as emphasized by the recent guidelines of the European Federation of Periodontology (Herrera et al. 2023). During these visits, clinicians are expected to identify risk factors, motivate and educate patients about oral home care, and detect early signs of peri‐implant inflammation. When biofilm accumulation or mucosal inflammation is identified, supportive care frequently requires the use of implant surface decontamination methods to re‐establish peri‐implant health.

Despite the critical role of bacterial biofilm removal, current evidence does not establish a definitive standard protocol that guarantees optimal effectiveness (Stiesch et al. 2023; Verket et al. 2023). Consequently, the decontamination methods employed, whether as a single therapy or in combination, may not always lead to complete disease resolution. For instance, a recent systematic review assessing the effectiveness of treatments for peri‐implant mucositis reported a resolution rate of 30% at 3 months, which dropped to 8%–16% at 6 months (Verket et al. 2023). Given the potential alterations caused by decontamination methods, a systematic review by Louropoulou et al. (2012) categorized non‐metal instruments as “implant‐safe” and suggested they might be the instruments of choice for treating implant surfaces. However, as previously noted, these instruments show limited efficacy in resolving plaque‐induced inflammation (Gennai et al. 2023; Verket et al. 2023), and the conclusions of this systematic review were largely based on SEM qualitative analysis at high risk of operator‐derived bias. More recent studies, quantitatively evaluating surface alterations following instrumentation, showed that there is a clear need for a new systematic review to better define the extent of surface alterations caused by decontamination methods.

The objective of this systematic review was to evaluate the impact of biofilm decontamination methods on the integrity of implant‐abutment surfaces. The PICO question was: on implants and abutments requiring biofilm removal (P), is there a method of implant surface decontamination (I) that has a smaller impact than others (C) in terms of implant surface alteration (O)?

## 2 Material and Methods

This systematic review was conducted according to the Preferred Reporting Items for Systematic Reviews and Meta‐Analyses Protocols (PRISMA) statement using the Population, Intervention, Comparison, and Outcome (PICO) method (Page et al. 2021). The protocol for this systematic review was registered on OSF (https://doi.org/10.17605/OSF.IO/MJ36N).

### 2.1 Search Strategy

Systematic searches of PubMed, Scopus, and Web of Science were conducted in September 2024 with no date limits. The specific search terms are provided in Appendix S1. Backward citation screening and forward citation chasing (Scopus, Web of Science) were additionally performed for all included studies.

### 2.2 Eligibility Criteria

Studies were included if they fulfilled the following criteria: studies which conducted (1) a quantitative analysis of implant or abutment surface after (2) using non‐surgical physical, mechanical, or chemical methods for implant surface decontamination. Only studies with full‐text availability and written in English were considered.

Studies were excluded if they: (1) applied more than one decontamination method in the same test arm; (2) analyzed the threaded/intraosseous region; (3) were qualitative‐only; (4) specifically evaluated peri‐implantitis treatment; (5) did not report roughness outcomes; (6) lacked a control (uninstrumented) surface; or (7) were reviews, conference abstracts, letters, duplicates, or other non‐original research.

### 2.3 Selection of Studies

The article selection process was carried out in two stages by two independent reviewers (LV and AG) with the aid of the Rayyan software. Screening decisions were masked until the end of the article selection process. In the first stage, the reviewers evaluated the titles and abstracts of the papers applying the eligibility criteria. Any disagreement was resolved through discussion, with a third reviewer (MM) arbitrating any unresolved disagreements. In the second stage, the same two independent reviewers conducted a full‐text screening of the papers, applying the eligibility criteria. All potentially relevant studies were recorded and evaluated for inclusion. Likewise, any disagreement was resolved through discussion, with unresolved disagreements arbitrated by a third reviewer (MM). All the reasons for exclusion were reported. If the full text was unavailable or the data were unextractable or unclear, corresponding authors were contacted via email and, when applicable, via ResearchGate. In both cases, a response from authors was waited for almost 1 month.

### 2.4 Data Extraction

Two authors (LV and AG) used a data extraction form and independently collected the following information from the included studies: (1) Study characteristics: title, authors' names, and published date. (2) Sample characteristics: implant materials and types of surfaces. (3) Intervention: decontamination methods and their application procedures. (4) Outcome: quantitative measurements. (5) Roughness parameter: parameter used, value of control and test groups, and *p*‐value if it is present. (6) Control group: no treatment.

Any disagreements between reviewers were resolved by discussion to reach a consensus or by consultation with a third reviewer (MM).

### 2.5 Quality Assessment

The risk of bias for all the included studies was independently assessed in duplicate by two reviewers (LV and AG). The Quality Assessment Tool for In Vitro Studies (QUIN) was used (Sheth et al. 2024).

## 3 Results

### 3.1 Study Selection

The systematic search returned 3808 references (1173 in Scopus, 1346 in Web of Science, 1289 in PubMed). After removing duplicates, 2345 studies were eligible for title screening. Following the screening of titles and abstracts, 2258 articles were excluded. Full‐text examination was then conducted on 87 articles, and finally 27 papers were included in this review. The references of included studies were also checked, leading to the identification of nine additional records, of which two were included in the review. This brought the total number of studies included in the review to 29. Figure S1 presents a flowchart depicting the screening and selection processes. The detailed PRISMA checklist can be found in Table S1.

### 3.2 Data Synthesis

The main characteristics of the 29 included studies are summarized in Table 1. Quantitative surface evaluation was performed using a profilometer (19 studies), a laser microscope (9 studies), or both (1 study). The primary parameters considered and reported by the studies were Ra (25 studies), Sa (8 studies), and Rz (8 studies). Specifically, Ra refers to the arithmetic mean roughness of the profile, Sa to the arithmetic mean height of the surfaces, and Rz to the maximum height of the profile. Other parameters, reported in the Table S2, were Rq (5 studies), Sku (4 studies), Sz (4 studies), Rmax (2 studies), Rt (2 studies), Sdr (2 studies), Pa (1 study), and St (1 study).

**TABLE 1:** Main characteristics of included studies.

Author, year	Material and surface	Decontamination methods	Instrumentation parameters	Measuring device	Measurement settings	Parameters measured
Batsukh et al. (2017)	Titanium discs Machined	Ultrasonic; Rubber cup; Laser; Chlorhexidine	Time and power of instrumentation were documented	Profilometer	Cut‐off 0.25 mm; evaluation length 2 mm	Ra
Bayrak et al. (2022)	Titanium discs Modified	Titanium curette; Titanium brush; Laser	Single operator, time, power, and orientation of instrumentation were documented	Profilometer	Cut‐off 0.25 mm; length assessment 1.25 mm	Ra
Bertoldi et al. (2017)	Titanium abutment Machined	Ultrasonic; Metal curette; Titanium curette.	Only time of instrumentation was documented	Profilometer	Range 350 μm	Ra
Biazussi et al. (2019)	Titanium disc Machined	Air‐polishing	Time and orientation of instrumentation were documented	Profilometer	Not reported	Ra
Cafiero et al. (2017)	Titanium implant necks Machined	Rubber cup; Rotary brush; Air‐polishing	Time, power, force, and orientation of instrumentation were documented	Profilometer	Not reported	Ra, Rz
Chun et al. (2017)	Titanium discs Machined	Metal scaler	Single operator, time, power, force, and orientation of instrumentation were documented	Microscope	100× magnification; 543 nm light source	Ra
Duarte et al. (2009)	Titanium discs Machined and Modified	Titanium curette; Plastic curette; Air‐polishing; Laser	Single operator, time of instrumentation was measured; no standardization for scaling force	Profilometer	10 mm stylus; cut‐off 0.8 mm; speed 0.1 mm/s	Ra
Ercan et al. (2014)	Titanium discs Modified	Laser	Time, orientation, and power of instrumentation were documented	Profilometer	Stylus radius 5 μm	Ra
Faccioni et al. (2021)	Titanium discs Machined and Modified	Ultrasonic	Single operator, time, power, and orientation of instrumentation were documented	Profilometer	500 × 500 μm	Ra
Fakhravar et al. (2012)	Titanium abutment Modified	Metal scaler; Plastic scaler	Single operator, time of instrumentation was measured	Profilometer	Stylus force 0.7 N; sample length 0.25 mm; tracing speed 0.5 mm/s	Ra
Gehrke et al. (2018)	Titanium‐nitride–coated implant abutment Machined	Plastic scaler; titanium curette; ultrasonic	Time and pressure of instrumentation was measured	Profilometer	Not reported	Sa
Huang et al. (2019)	Titanium round discs; zirconium round discs Machined	Ultrasonic; titanium curette; plastic curette; Air‐polishing	Single well‐trained operator, power, and orientation of instrumentation were documented	Profilometer	Not reported	Ra
Hui et al. (2021)	Titanium disc Machined and Modified discs	Cold atmospheric plasma; Air‐Polishing	Single operator, time, power, and distance of instrumentation was measured	Profilometer	Not reported	Sa
Khalil and Sakr (2023)	Titanium discs Modified	Laser	Time and power of instrumentation were documented	Profilometer	Cut‐off 0.8 mm; speed 0.5 mm	Ra
Kim et al. (2019)	Titanium discs Machined and Modified	Nylon brush; Metal brush	2 different operators; no standardized protocol	Microscope	Cut‐off length 0.04 mm; z‐sections 0.80 μm.	Ra, Rz, Sa
Kim et al. (2020)	Titanium discs Modified	Laser	Time and power of instrumentation were documented	Microscope	Not reported	Ra
Kister et al. (2017)	Titanium discs Machined	Ultrasonic; Metal curette; Plastic curette; Rubber cup; Rotary brush; Air‐polishing	Time, power, force, and orientation of instrumentation were documented	Profilometer	2 μm diamond stylus	Ra
Lang et al. (2016)	Titanium discs; titanium‐zirconium discs; zirconium discs Machined	Titanium curette; Plastic curette; Titanium brush; Laser	Single operator, forces not calibrated or measured	Profilometer	Scan length 4.4 mm	Ra
Lee et al. (2023)	Titanium discs Machined and Modified	Laser	Time and power of instrumentation were documented	Microscope	10× magnification	Ra, Sa
Li et al. (2022)	Titanium discs Modified	Laser	Frequency, wavelength, and power of instrumentation were documented	Microscope	2000×, 5000×, and 10,000× magnification	Sa
Park et al. (2012)	Titanium discs Machined and Modified	Ultrasonic; Toothbrush	Single operator, time, power, force, and orientation of instrumentation were documented	Microscope	Cut‐off length 0.04 mm; z‐sections 0.80 μm.	Ra, Rz, Sa
Park et al. (2013)	Titanium discs Modified	Ultrasonic; Toothbrush	Single operator, time, power, force, and orientation of instrumentation were documented	Microscope	Cut‐off length 0.04 mm; z‐sections 0.80 μm	Ra, Rz, Sa
Park et al. (2015)	Titanium discs Machined and Modified	Titanium brush	Single operator, time, power, and force of instrumentation were documented	Profilometer and Microscope	Cut‐off length 0.04 mm; z‐sections 0.80 μm.	Ra, Rz, Sa
Sahrmann et al. (2021)	Titanium discs Machined and Modified	Ultrasonic	Time, power, and orientation of instrumentation were documented	Profilometer	Not reported	Ra, Rz
Sawase et al. (2005)	Titanium coated and titanium pure discs Machined	Ultrasonic; Metal scaler; Plastic scaler; Rotary brush; toothbrush	Single skilled operator, power, time, and force were documented	Profilometer	Traverse speed 0.1 mm/s	Ra
Stübinger et al. (2008)	Zirconia discs Machined	Laser	Time, energy, and frequency of instrumentation were documented	Microscope	Not reported	Sa
Tan et al. (2022)	Zirconia discs Modified	Titanium curette; Plastic curette; Air‐Polishing; Ultrasonic	Time and power of instrumentation were documented	Microscope	Random measurement	Sa
Toma et al. (2018)	Titanium discs Modified	Plastic curette; Titanium brush; Air‐polishing; burs	Time, power, and orientation of instrumentation were documented	Profilometer	Stylus diameter 7 μm	Ra, Rz
Unursaikhan et al. (2012)	Titanium discs Machined	Ultrasonic; Plastic curette	Single operator, time, power, and orientation of instrumentation were documented	Profilometer	Not reported	Ra, Rz

Four different types of surfaces were identified and grouped into the following four categories: modified titanium (including sand‐blasted and acid‐etched surfaces, sand‐blasted, resorbable blast material, resorbable blast texturing, Inicell, anatase‐coated, laser micropatterned, and femtosecond laser) in seventeen studies; machined titanium (polished, nitride‐coated, smooth) in nineteen studies; zirconia in four studies; and mixed surfaces (an alloy containing 85% titanium and 15% zirconia) in one study. Most of the analyzed samples were disks, although abutments and implant necks were also included. The control group consisted of untreated surfaces or surfaces analyzed pre‐treatment.

Included mechanical instruments were air polishers (erythritol, glycine, sodium bicarbonate), brushes (metal, nylon, titanium), toothbrushes, rubber cups, curettes (metal, plastic, titanium), scalers (metal, plastic), and ultrasonic devices (bronze, carbon, copper, metal, plastic, resin, titanium). Physical instruments comprised lasers (diode, Er:Cr:YSGG, Er:YAG, Nd:YAG, GaAlAs, CO_2_), and cold atmospheric plasma. Chemical instruments included chlorhexidine.

Given the marked heterogeneity in study designs, surfaces, measurement methods, roughness parameters, and the large number of decontamination methods, no meta‐analysis was undertaken. For all comparisons we calculated the change score (=mean_test_ − mean_control_, μm), which was not reported in the primary studies and has been added to our extraction. Tables S4–S7 present the extracted metrics together with. In the main manuscript, Tables 2, 3, 4, 5 provide a concise synthesis by surface and roughness parameter: when an instrument was evaluated only once (single study/condition), we report the mean and the difference between standard deviations (SD_test_ − SD_control_); when an instrument was evaluated across multiple studies or experimental conditions, we report the weighted mean (weights equal to the number of test measurements), the mean SD difference, and the observed range (minimum–maximum).

**TABLE 2:** Surface roughness values on modified titanium surfaces (𝛿, μm).

Instrument	Type	Ra	Sa	Rz
Air‐Polishing	Erythritol	NA	0.35; NA S/C: 1/1	NA
	Glycine	−0.34; ↑ 0.03 S/C: 1/1	NA	−0.57; ↓ 0.07 S/C: 1/1
	Sodium Bicarbonate	−0.01; 0.0 S/C: 1/1	NA	NA
Brush	Metal	−0.28; ↓ 0.04 (−0.39;‐0.17) S/C: 1/2	−0.19; ↑ 0.07 (−0.33;‐0.04) S/C: 1/2	−1.21; ↓ 0.16 (−1.55;‐0.86) S/C: 1/2
	Nylon	−0.19; ↓ 0.09 (−0.31;−0.07) S/C: 1/2	‐0.07; ↑ 0.01 (−0.27;0.13) S/C: 1/2	−0.47; ↓ 0.37 (−0.74;‐0.19) S/C: 1/2
	Titanium	−0.37; ↑ 0.02 (−0.63;0.04) S/C: 3/4	NA	NA
Cold Atmospheric Plasma		NA	0.47; NA S/C: 1/1	NA
Curette	Metal	0.02; ↑ 0.24 S/C: 1/1	NA	NA
	Plastic	−0.02; ↑ 0.06 (−0.04;0.0) S/C: 2/2	NA	NA
	Titanium	−1.03; ↑ 0.04 S/C: 1/1	NA	NA
Laser	Diode	0.14; ↑ 0.05 (−0.19;0.96) S/C: 4/14	−0.2; 0 (−0.28;‐0.12) S/C: 1/2	NA
	Er,Cr:YSGG	0.17; ↑0.01 (−0.33;0.75) S/C: 3/12	0.13; NA (0.07;0.18) S/C: 1/2	NA
	Er:YAG	−0.02; ↓0.64 S/C: 1/1	NA	NA
	Nd:YAG	NA	0.04; ↑ 0.08 (0.02;0.05) S/C: 1/2	NA
Toothbrush		−0.44; ↑ 0.05 (−0.71;‐0.17) S/C: 2/2	−0.26; ↑ 0.13 (−0.7;0.19) S/C: 2/2	−1.81; ↑ 0.02 (−2.85;‐0.77) S/C: 2/2
Ultrasonic	Carbon	−0.18; ↑ 0.08 (−0.34;0.31) S/C: 2/3	0.32; ↑ 0.11 S/C: 1/1	−1.73; ↓ 0.24 (−2.18;‐0.24) S/C: 2/3
	Metal	−0.58; ↑0.14 (−0.8;‐0.2) S/C: 3/6	−0.30; ↑ 0.08 (−0.58;‐0.05) S/C: 2/4	−3.18; ↑ 0.30 (−3.99;1.18) S/C: 3/6
	Plastic	−0.32; ↑0.1 (−0.49;‐0.08) S/C: 3/5	−0.10; ↑ 0.06 (−0.2;0.1) S/C: 2/3	−2.54; ↑ 0.13 (−3.3;‐0.94) S/C: 3/5
	Resin	0.02; ↑0.05 (0.0;0.03) S/C: 1/2	NA	−0.41; ↑ 0.31 (−0.43;‐0.39) S/C: 1/2
	Titanium	−0.54; ↑0.08 (−0.61;‐0.22) S/C: 2/4	NA	1.45; ↑ 0.63 (0.7;2.19) S/C: 1/2

*Note:* Δ = mean_test_‐mean_control_ (μm; negative = decrease). Single evaluation: mean Δ and ΔSD (SD_test_‐SD_control_). Multiple evaluations: weighted mean Δ (weights = number of tests), mean ΔSD, and Δ range (min–max). S/C: number of studies/number of conditions (distinct experimental condition within a study. e.g., different pressures, power, or surfaces). **↑:** SD_test_ > SD_control_. **↓:** SD_test_ < SD_control_.

Abbreviation: NA, Not Available.

### 3.3 Quality of Studies

An overview of the QUIN quality assessment of the included studies is provided in Table S3. The overall risk of bias was low in 3 studies, high in 3 studies and moderate in 23. The parameters that received the lowest scores were related to randomization, the use of blinding, a detailed explanation of sample size determination, and information about the outcome assessor.

### 3.4 Surface Analysis

#### 3.4.1 Ra

##### 3.4.1.1 Modified Titanium Surfaces

The results of the fourteen included studies that evaluated the Ra parameter on modified titanium surfaces are summarized in Table 2, with details in Table S4. Ra of untreated or pre‐treated surfaces ranges from 0.177 to 2.81 μm. While some studies suggest that plastic curettes (Duarte et al. 2009) and resin ultrasonic inserts (Sahrmann et al. 2021) do not alter surface topography, others report conflicting results. Toma et al. (2018) showed a decrease in surface roughness, whereas Sahrmann et al. (2021), on a different modified surface with a similar baseline Ra, reported an increase. Nylon and metal polishing brushes, Saeshin laser, toothbrush, titanium curette, air powder, and ultrasonic inserts in metal, plastic, and titanium consistently reduce the initial roughness, as reported by all studies that analyzed these instruments. On the other hand, a homogeneous increase in Ra is induced by metal curette (Bayrak et al. 2022; Duarte et al. 2009; Faccioni et al. 2021; Kim et al. 2020, 2019; Park et al. 2013, 2012; Sahrmann et al. 2021; Toma et al. 2018). However, two different air powders and metal curettes were analyzed only by a single study (Duarte et al. 2009; Toma et al. 2018).

For titanium polishing brushes and carbon ultrasonic inserts, some studies show a decrease in the Ra parameter (Bayrak et al. 2022; Park et al. 2015; Sahrmann et al. 2021; Toma et al. 2018), while others observe the opposite effect (Park et al. 2013, 2015). The results regarding lasers appear to be more heterogeneous. Except for the Saeshin laser, other laser types such as Er,Cr:YSGG, Er:YAG, Epic10, and K2Mobile report both types of results. Specifically, within the same studies, there are results indicating both a reduction and an increase in the initial Ra parameter (Bayrak et al. 2022; Duarte et al. 2009; Ercan et al. 2014; Khalil and Sakr 2023; Kim et al. 2020; Lee et al. 2023).

##### 3.4.1.2 Machined Titanium Surfaces

The results of the eighteen included studies evaluating Ra on treated titanium surfaces are summarized in Table 3 (details in Table S5). Ra of untreated or pre‐treated surfaces ranges from 0.017 to 1.182 μm.

**TABLE 3:** Surface roughness values on machined titanium surfaces (𝛿, μm).

Instrument	Type	Ra	Sa	Rz
Air‐Polishing	Erythritol	NA	−0.21; NA S/C: 1/1	NA
	Glycine	−0.03; ↓ 0.01 (−0.07;0.0) S/C: 3/4	NA	−1.63; ↓ 0.12 (−2;‐1.25) S/C: 1/2
	Sodium Bicarbonate	0.05; ↑ 0.03 (−0.01;0.1) S/C: 3/4	NA	NA
Brush	Metal	0.21; ↑ 0.04 S/C: 1/1	0.28; ↑ 0.11 S/C: 1/1	0.87; ↑ 0.08 S/C: 1/1
	Nylon	0.06; ↑ 0.02 (0–0.35) S/C: 3/5	0.35; ↑ 0.11 S/C: 1/1	−0.05; ↓ 0.21 (−0.90;0–95) S/C: 2/3
	Titanium	0.09; ↓ 0.11 (−0.27;0.05) S/C: 2/3	−0.39; ↓ 0.4 S/C: 1/1	−1.07; ↓ 0.92 S/C: 1/1
Chlorhexidine	1 mL of 0.12%	−0.01; ↓ 0.03 S/C: 1/1	NA	NA
Cold Atmospheric Plasma		NA	0.28; NA S/C: 1/1	NA
Cup	Rubber	0.05; ↑ 0.02 (−0.04;0.1) S/C: 3/5	NA	−0.80; ↓ 0.24 (−1;‐0.6) S/C: 1/2
Curette	Metal	0.21; ↑ 0.05 (0.03;0.48) S/C: 3/4	NA	NA
	Plastic	−0.16; ↑ 0.01 (−0.48;0.05) S/C: 5/7	NA	−0.11; ↓ 1.19 S/C: 1/1
	Titanium	0.37; ↑ 0.04 (−0.04;0.62) S/C: 4/5	−0.05; ↓ 0.01 S/C: 1/1	NA
Laser	Diode	0.15; ↑ 0.01 (0.08;0.17) S/C: 2/2	0.06; NA S/C: 1/1	NA
	Er,Cr:YSGG	0.45; 0 S/C: 1/1	1.58; NA S/C: 1/1	NA
	Er:YAG	0.05; ↑ 0.04 S/C: 1/1	NA	NA
	GaAlAs	0; ↓ 0.02 S/C: 1/1	NA	NA
Scaler	Metal	0.02; NA (0.02) S/C: 2/3	NA	NA
	Plastic	0.01; NA (0;0.01) S/C: 3/4	−0.06; ↓ 0.01 S/C: 1/1	NA
Toothbrush		−0.03; ↓ 0.10 (−0.27;0.05) S/C: 2/3	−0.29; ↓ 0.38 S/C: 1/1	−0.15; ↓ 0.29 S/C: 1/1
Ultrasonic	Bronze	0.1; 0 S/C: 1/1	NA	NA
	Carbon	0; 0 (−0.05;0.08) S/C: 2/3	NA	−0.03; ↓ 0.01 (−0.44;0.38) S/C: 1/2
	Metal	0.7; ↓ 0.02 (−0.05;5.30) S/C: 8/18	1.16; ↑ 0.29 (0.01;3.3) S/C: 2/3	0.94; ↑ 0.13 (0.1;2.28) S/C: 3/10
	Plastic	−0.01; ↓ 0.06 (−0.06;0.01) S/C: 4/7	−0.11; ↓ 0.31 (−0.15;‐0.06) S/C: 1/2	0.22; ↓ 0.28 (−0.22;0.8) S/C: 2/4
	Resin	−0.02; ↓ 0.01 (−0.03;0) S/C: 1/2	NA	0.01; ↓ 0.03 (−0.26;0.28) S/C: 1/2
	Titanium	0.18; ↑ 0.14 (−0.06;0.26) S/C: 2/4	NA	1.45; ↑ 0.63 (0.7;2.19) S/C: 1/2

*Note:* Δ = mean_test_ − mean_control_ (μm; negative = decrease). Single evaluation: mean Δ and ΔSD (SD_test_ − SD_control_). Multiple evaluations: weighted mean Δ (weights = number of tests), mean ΔSD, and Δ range (min–max). S/C: number of studies/number of conditions. **↑**: SD_test_ > SD_control_. **↓**: SD_test_ < SD_control_.

Abbreviation: NA, Not Available.

Significant variability was observed, with some instruments increasing Ra in one study and decreasing it in another, leading to inconsistent and conflicting findings overall.

Glycine and sodium bicarbonate powders were associated with both increased (Biazussi et al. 2019; Duarte et al. 2009; Kister et al. 2017) and decreased Ra (Cafiero et al. 2017; Huang et al. 2019; Kister et al. 2017), while only Cafiero et al. (2017) reported glycine maintaining surface topography. Titanium curettes typically increased Ra (0.1–1.71 μm), with one study reporting a reduction of 0.04 μm (Bertoldi et al. 2017). Rubber cups showed both increases (Cafiero et al. 2017; Kister et al. 2017) and reductions (Batsukh et al. 2017; Kister et al. 2017). Titanium brushes yielded inconsistent results: one study reported a 0.28 μm increase (Lang et al. 2016), another a 0.33 μm reduction or no change (Park et al. 2013, 2015). Ultrasonic carbon, titanium, and metal inserts mostly increased Ra, though reductions were also observed (Faccioni et al. 2021; Sahrmann et al. 2021; Unursaikhan et al. 2012). Metal US inserts showed the highest agreement, with roughening (range 0.07–5.3 μm) observed in 15 out of 17 data values. Plastic inserts mainly reduced Ra, though minimal increases (0.01 μm) were reported (Kister et al. 2017; Sahrmann et al. 2021). Lasers predominantly caused roughening, except for GaAlAs, which reduced Ra (0.008 μm) (Batsukh et al. 2017).

Both plastic scalers and toothbrushes generally increased Ra; however, one study reported no change for plastic scalers (Sawase et al. 2005), while another found a reduction of 0.27 μm for toothbrushes (Park et al. 2012). Only nylon brushes, metal scalers, and metal curettes consistently increased Ra across multiple studies (0.19–2.04 μm). Single studies suggest metal brushes and bronze/copper ultrasonic inserts increase Ra, while resin inserts and chlorhexidine reduce it (Batsukh et al. 2017; Chun et al. 2017; Sahrmann et al. 2021).

##### 3.4.1.3 Zirconia

Only two studies evaluated zirconia surfaces (Table 4; Table S6), with Ra values ranging from 0.07 to 0.9 μm in the control group and from −0.05 to 0.06 μm in the test group. Glycine powder does not alter the initial Ra value. Plastic curettes consistently increase surface roughness by 0.3–0.4 μm, as does the carbon ultrasonic insert, which causes a roughening of 0.1 μm. The laser, on the other hand, reduces roughness by 0.04 μm. However, results for titanium curettes are less consistent: Huang et al. (2019) report a roughening of 0.6 μm, while Lang et al. (2016) observe no change in one case and a decrease in Ra in another.

**TABLE 4:** Surface roughness values on zirconia surfaces (𝛿, μm).

Instrument	Type	Ra	Sa
Air‐Polishing	Glycine	0; ↑ 0.01 S/C: 1/1	0.18; NA S/C: 1/1
Curette	Plastic	0.03; ↑ 0.03 (0.03;0.04) S/C: 2/3	0.2; NA S/C: 1/1
	Titanium	−0.01; ↑ 0.15 (−0.05;0.06) S/C: 2/3	0.24; NA S/C: 1/1
Laser	Diode	−0.04; ↑ 0.02 S/C: 1/1	NA
	CO_2_	NA	0.53; ↑ 0.37 (0.28;0.87) S/C: 1/5
Ultrasonic	Carbon	0.01; ↑ 0.01 S/C: 1/1	NA
	Metal	NA	0.44; NA S/C: 1/1

*Note:* Δ = mean_test_ − mean_control_ (μm; negative = decrease). Single evaluation: mean Δ and ΔSD (SD_test_ − SD_control_). Multiple evaluations: weighted mean Δ (weights = number of tests), mean ΔSD, and Δ range (min–max). S/C: number of studies/number of conditions. **↑**: SD_test_ > SD_control_. **↓**: SD_test_ < SD_control_.

Abbreviation: NA, Not Available.

##### 3.4.1.4 Mixed

One study (Lang et al. 2016) analyzed titanium‐zirconium alloy surfaces (Table 5; Table S7), reporting the highest Ra increase for titanium curettes, at 0.47 μm and 0.39 μm. Following that, titanium polishing brushes increased roughness by 0.38 μm, and the laser by 0.28 μm. For plastic curettes, the study found a smaller surface roughening, ranging from 0.08 μm to 0.1 μm.

**TABLE 5:** Surface roughness values on mixed surfaces (𝛿, μm).

Instrument	Type	Ra
Brush	Titanium	0.34; ↓ 0.02 S/C: 1/1
Curette	Plastic	0.09; ↓ 0.05 (0.08;0.1) S/C: 1/2
	Titanium	0.43; ↑ 0.11 (0.39;0.47) S/C: 1/2
Laser	Diode	0.28; ↑ 0.03 S/C: 1/1

*Note:* Δ = mean_test_ − mean_control_ (μm; negative = decrease). Single evaluation: mean Δ and ΔSD (SD_test_ − SD_control_). Multiple evaluations: weighted mean Δ (weights = number of tests), mean ΔSD, and Δ range (min–max). S/C: number of studies/number of conditions. **↑**: SD_test_ > SD_control_. **↓**: SD_test_ < SD_control_.

Abbreviation: NA, Not Available.

#### 3.4.2 Sa

##### 3.4.2.1 Modified Titanium Surfaces

The results of the seven included studies that evaluated the Sa parameter on modified titanium surfaces are summarized in Table 2 (details in Table S4). Sa of untreated or pre‐treated surfaces ranges from 1.48 to 2.81 μm.

Ultrasonic metallic and plastic tips, titanium brush and metal brush show a general reduction in the Sa parameter (Kim et al. 2019; Park et al. 2012, 2015), with only one exception for ultrasonic plastic tips which report an increase of 0.1 μm (Park et al. 2013).

Lasers, as Nd:YAG and Er,Cr:YSGG, caused an increase of initial roughness from 0.01 μm to 0.18 μm (Li et al. 2022). Only Diode lasers induced a decrease in Sa by 0.28 μm (Lee et al. 2023).

For nylon brush and toothbrush studies reports heterogeneous results; both instruments induce an increase and a reduction of surface roughness (Kim et al. 2019; Park et al. 2013, 2012).

Only one study analyzes the impact of cold atmospheric plasma spray and reports an increase in Sa by 0.47 μm (Hui et al. 2021).

No instruments maintained Sa parameters unaltered. Additionally, the major increase was obtained following the use of erythritol powder (0.87 μm) (Hui et al. 2021), while the major reduction was obtained using a toothbrush (0.7 μm) (Park et al. 2013).

##### 3.4.2.2 Machined Titanium Surfaces

Six included studies report the values of the Sa parameter after instrumentation on machined titanium surfaces, which are summarized in Table 3 (details in Table S5). Sa parameters of these surfaces in the control group ranged from 0.19 to 1.19 μm.

An homogeneous increase in Sa parameter is reported following the use of lasers, as Diode laser and Er,Cr:YSGG, ultrasonic metal and titanium tips, and nylon and metal brush (Gehrke et al. 2018; Kim et al. 2019; Lee et al. 2023; Park et al. 2012) Specifically, ultrasonic metal tips show the highest increase (3.3 μm) compared to all results (Gehrke et al. 2018). On the other hand, ultrasonic plastic tips, titanium curette and plastic scaler induced a decrease in surface roughness (Gehrke et al. 2018; Park et al. 2012).

Toothbrush decreased Sa parameter by 0.29 μm (Park et al. 2012), and similarly titanium brush induced the highest reduction in Sa (0.41 μm) (Park et al. 2015) compared to all other instruments.

##### 3.4.2.3 Zirconia Surfaces

Zirconia surfaces were analyzed only by two studies (Stübinger et al. 2008; Tan et al. 2022).

CO_2_ lasers, plastic and titanium curettes, glycine powder and ultrasonic metal tips increase homogeneously surface roughness. Specifically, the highest is induced by the ultrasonic metal tip (0.44 μm), then the lowest is by glycine powder (0.18 μm) (Table 4; Table S6).

#### 3.4.3 Rz

##### 3.4.3.1 Modified Titanium Surfaces

Rz parameter was analyzed by six studies on modified titanium surfaces, and it ranges in the control group from 6.71 to 9.79 μm (Table 2; Table S4).

All instrumentation decreases Rz values, with the only exception of the plastic curette, whose use induced an increase of 0.19 μm (Toma et al. 2018). On the other hand, the maximum decrease of this parameter was shown following the use of the ultrasonic metal tip (3.99 μm) (Sahrmann et al. 2021).

Results near the maximum reduction value regard ultrasonic metal and titanium tips, and toothbrush (Park et al. 2013, 2012; Sahrmann et al. 2021). A lower reduction was reported for ultrasonic plastic and resin tips (from 0.94 to 0.39 μm) (Park et al. 2013, 2012; Sahrmann et al. 2021) and for glycine powder (1.55 μm to 0.57 μm) (Toma et al. 2018).

Polishing brushes in nylon, metal, and titanium decrease in Rz with a range from 1.55 to 0.19 μm (Kim et al. 2019; Park et al. 2015; Toma et al. 2018).

##### 3.4.3.2 Machined Titanium Surfaces

Surface roughness of machined titanium surface in Rz parameter was evaluated by six studies (Table 3; Table S5). The Rz of the control group ranged from 0.02 to 3.05 μm.

For machined titanium surface results are heterogeneous, except for a general decrease induced by glycine powder, rubber cup, titanium brush and plastic curette (Cafiero et al. 2017; Park et al. 2015; Unursaikhan et al. 2012), and a general increase after using an ultrasonic metal and titanium tip (Park et al. 2012; Sahrmann et al. 2021; Unursaikhan et al. 2012).

Ultrasonic resin, carbon and plastic tips mostly increase the Rz parameter, even if some results show a decrease after their use (Sahrmann et al. 2021). Similarly, the nylon brush caused both an increase and a decrease in surface roughness (Cafiero et al. 2017; Kim et al. 2019).

Specifically, the highest increase was shown using ultrasonic metal tips (2.28 μm) (Cafiero et al. 2017; Sahrmann et al. 2021) while the major decrease was shown using glycine powder (2 μm) (Cafiero et al. 2017).

## 4 Discussion

This systematic review evaluated the effects of biofilm decontamination methods on the trans‐mucosal portion of dental implants, to assess if different tools and procedures are able to fully preserve the initial topography of implant surfaces.

The analysis of the included in vitro quantitative studies highlights significant heterogeneity in results, which limits the ability to identify a universally safe instrumentation tool. Notably, the findings indicate that all instruments may compromise the integrity of titanium and titanium‐zirconia implant surfaces. Evidence for zirconia is confined to two studies showing fewer surface alterations; while this may relate to higher hardness, elastic modulus, and wear resistance, the limited dataset restricts inference and generalizability.

Several factors may have contributed to those conflicting results. One key factor is the variation in surface measurement methodologies. Surface roughness analysis was carried out with either profilometers (optical, laser, and contact) or microscopes (confocal, optical, laser, and scanning electron), each one of them carrying some strengths and limitations (Demircioglu and Durakbasa 2011; Durakbasa et al. 2011). For instance, mechanical contact profilometers while offering a wider measuring range, may be less accurate due to stylus sensitivity and contamination. On the other hand optical methods, may provide high resolutions, but may overestimate roughness on modified surfaces and produce high reflectance in smooth ones (Wennerberg and Albrektsson 2000). These methodological differences may theoretically lead to conflicting results when evaluating the same surface conditions. Among the studies included in this review, only Park et al. (2015) utilized both confocal microscopy and a profilometer to evaluate the effect of a titanium brush on machined and sandblasted implant surfaces. The authors reported only minimal discrepancies in the results. Specifically, for sandblasted surfaces, the difference in Ra between control and test groups was 0.04 μm when measured with a profilometer and −0.04 μm when measured by microscopy. For smooth surfaces, the microscope detected a difference of −0.33 μm between control and test groups, while the profilometer indicated no alterations. Despite these differences, the authors concluded that both tools are valid, as the variations were not statistically significant. The results presented by Park et al. (2015) should be interpreted with caution, as they were limited to the use of a titanium brush on two different surfaces. It can be hypothesized that different devices might produce surface alterations with distinct morphologies on different implant surfaces. Given these uncertainties, in the absence of consensus on the most appropriate roughness‐measurement approach, inferences across methods, and studies should be made cautiously.

A second issue that may have an impact on surface roughness is the wide variability of the decontamination protocols applied across the studies included in terms of force, power, angulation, distance, number of strokes, applied pressure, and application time. However, some studies reported that surface roughness (Ra and Rz) was not influenced either by the use of a 45° or 15° angulation of ultrasonic tip (Unursaikhan et al. 2012) nor by the number of strokes with a curette or by the pressure of air‐abrasive powder (Cafiero et al. 2017; Lang et al. 2016). Conversely, in the case of Er,Cr:YSGG lasers, critical factors affecting surface alterations include the distance, power, and application time (Ercan et al. 2014). Overall, the limited number of studies on application parameters, combined with the complexity of the variables involved, hinders cross‐study comparisons and may even preclude them.

A third possible source of heterogeneity relates to roughness parameters. The Ra parameters, one of the most widely used, represent the mean roughness value of a surface profile. However, it does not provide any information about spatial structure and outlier points (Taylor et al. 2005). The Rz parameters, which consider the five highest peaks and the five lowest valleys, offer better insights into outlying features (Demircioglu and Durakbasa 2011). Nonetheless, both Ra and Rz parameters focus on two‐dimensional profiles, whereas Sa and Sz evaluate similar properties but within a three‐dimensional area (Wennerberg and Albrektsson 2010).

In addition to these, several other parameters have been proposed in the literature such as Sdr parameters that quantify the developed interfacial area ratio, reflecting the relative increase in surface area compared to a flat baseline (Dohan Ehrenfest et al. 2010). As an expression of increased roughness, Sdr could be useful for exploring the relationship between surface changes and biofilm adhesion. This review, focused on the Ra, Sa, and Rz parameters, as they are widely used and well‐documented while other parameters were included in Appendix S1.

The outcome may also be influenced by the hardness, the quality or type of implant material, strictly linked to the kind of alloy or brand used. However, there are currently no studies that specifically evaluate how different instruments, used in the same manner, interact with various titanium or zirconia alloys.

A major critical point is understanding the potential biological implications of these surface alterations. Current evidence on the impact of surface roughness on bacterial colonization of implant surfaces remains limited. One of the most frequently cited studies reported that maintaining an Ra value below 0.2 μm may minimize differences in bacterial composition (Bollen et al. 1996). Similarly, Rimondini et al. (1997) proposed that surfaces with Ra < 0.088 μm and Rz < 1.027 μm could effectively reduce bacterial colonization. In the studies included in this systematic review, few implant surfaces are manufactured with roughness values below this threshold. Specifically, only 45% of control machined surfaces exhibited Ra values < 0.2 μm. Among these, slightly more than half exceeded this threshold after instrumentation with various decontamination methods, including sodium bicarbonate air‐powder, rubber cup, plastic, titanium and metal curettes, metal scalers, Er:YAG lasers, and metal ultrasonic tips. The absence of established thresholds and the lack of knowledge regarding the ability to quantify the impact of changes in roughness parameters on bacterial growth make data interpretation highly challenging.

Modified implant surfaces have been shown to positively influence osteogenic cell differentiation and improve osseointegration more effectively than smooth surfaces (Calciolari et al. 2018; Deng et al. 2015; Wen et al. 1996). However, although these surfaces are designed to promote osteointegration, when exposed due to the pathological process, they may instead facilitate bacterial adhesion. Rough surfaces may in fact promote greater bacterial colonization compared to smooth surfaces (Teughels et al. 2006), likely due to the ability of bacteria to adhere to irregular areas and niches. At the same time, topographical features larger than bacterial size may be perceived by bacteria as flat areas, whereas roughness on a scale compatible with bacterial dimensions significantly increases potential adhesion (Li et al. 2022). In addition to surface roughness and topography, other factors such as surface free energy, wettability, and chemical properties also contribute to influencing bacterial adhesion (Song et al. 2015).

Few studies included in this review analyzed the clinical implication of surface modifications resulting from decontamination procedures. Toma et al. (2018) reported a reduction in *S. gordonii* colonization after using a titanium brush and glycine air‐powder, attributed to a decrease in surface roughness. Similarly, Duarte et al. (2009) observed a reduction in *S. sanguinis* colonization after employing a metal curette. Conversely, no significant difference in bacterial colonization was observed when using a plastic or metal curette and laser Er:YAG. However, some conflicting results have been reported for machined surfaces. Batsukh et al. (2017) observed increased bacterial colonization after using metal ultrasonic tips, while Huang et al. (2019) reported similar findings for titanium curette. For zirconia surfaces, various instruments—including plastic and metal curette, carbon, stainless steel, and metal ultrasonic tips, and glycine air‐powder systems—did not significantly increase bacterial colonization (Huang et al. 2019; Tan et al. 2022). Notably, only two out of five studies included in the present systematic review reported a significantly increased biofilm adhesion following the use of metal instruments on smooth titanium surfaces.

Thus, the current lack of knowledge regarding the potential implications of bacterial adhesion following instrumentation prevents a clear understanding of the role of decontamination methods in bacterial regrowth. Although it seems reasonable that increasing roughness, as a result of decontamination methods, may actually enhance bacterial adhesion predisposing to develop peri‐implant diseases, no supporting evidence is available. Instead, studies focusing on the efficacy of decontamination procedures failed to report an increase in the incidence of peri‐implant diseases due to instrumentation‐induced surface damage. For instance, a RCT study with a 1‐year follow‐up period found no significant differences in probing depth or bleeding on probing among different decontamination protocols (Ziebolz et al. 2017).

These findings underscore the need for further in vivo research to better understand the clinical implications of surface modifications caused by decontamination methods, particularly concerning their potential contribution to the development of biological complications as peri‐implant disease.

The effects of decontamination methods on implant surface may have a certain parallelism with what is induced by implantoplasty used in peri‐implantitis therapy. Both procedures may lead to surface alteration. In terms of surface roughness induced by implantoplasty, some studies report final Ra parameters below 0.5 μm (Camps‐Font et al. 2023; Costa‐Berenguer et al. 2018), while others find values exceeding 0.3 μm (Ramel et al. 2016; Tsampli et al. 2024). Therefore, this procedure does not achieve Ra values below the threshold of bacterial colonization previously reported by Rimondini et al. (1997). Nonetheless, the literature consistently recognizes implantoplasty as an effective treatment to reduce surface roughness and, consequently, bacterial adhesion (Azzola et al. 2020). Supporting this, a recent systematic review concluded that implantoplasty does not appear to be associated with any mechanical or biological risks in the short to mid‐term (Stavropoulos et al. 2019). However, decontamination methods, such as implantoplasty, may also alter the biocompatibility of implant materials due to the release of particles and ions (Louropoulou et al. 2015).

Given the heterogeneity of the evidence and the absence of clinically validated thresholds, surface changes should not serve as a stand‐alone criterion. The choice of decontamination methods should be driven primarily by efficacy, with such changes weighed against case‐specific considerations. This does not imply that increased roughness or other alterations are irrelevant; however, in the absence of clear guidance from the literature, they could be regarded as secondary to achieving disease control. These case‐specific considerations may include the location and nature of the deposits (e.g., supramarginal vs. submarginal, coronal vs. apical) and their type (e.g., plaque, calculus, or residual cement). For example, residual cement is particularly difficult to remove and has been detected deep within the mucosal tunnel, more frequently in peri‐implantitis than in mucositis (Montevecchi et al. 2025), and may require dedicated tools. Accordingly, decontamination modalities should be tailored to the clinical scenario, with the dual aim of maximizing efficacy while preserving the original implant topography as far as possible.

This systematic review highlights the following limitations: first, the high heterogeneity among studies, which makes it difficult to identify clear clinical implications; second, the limited available evidence regarding surface roughness parameters that does not allow for a comprehensive interpretation of surface alterations; third, the region of the implant threads was not considered because its exposure is typically disease‐related and often requires a distinct decontamination approach; fourth, combined‐intervention studies were excluded to isolate method‐specific effects for comparative choice, which may limit generalizability because methods may be used in combination in clinical practice. Further studies should investigate the correlation between surface roughness and clinically relevant outcomes. This approach could help determine whether, how and to what extent different types of surface damage may influence, for instance, bacterial colonization and peri‐implant health.

In conclusion, our results indicate that the methods used for implant surface decontamination may alter the integrity and roughness of dental implant and abutment surfaces. All available studies were conducted in vitro, with no clinical or animal model data currently available, which limits the generalizability and clinical applicability of the findings. The impact of these alterations on peri‐implant health and long‐term clinical outcomes remains uncertain.

Despite the challenges in identifying safe instruments and methods for dental implant maintenance, current evidence highlights the essential role of supportive peri‐implant care in ensuring implant survival and success (Berglundh et al. 2019; Monje et al. 2016, 2017). In this context, the present review did not identify any method that can be regarded as universally surface‐safe, nor does it support prescriptive recommendations. Until higher‐quality clinical evidence is available, the choice of decontamination methods should be individualized and documented, taking into account case‐specific factors (e.g., deposit location and type, access), procedural feasibility, and the possibility of surface alteration.

## Author Contributions

**Leoluca Valeriani:** conceptualization, methodology, data curation, visualization, writing – original draft, investigation, writing – review and editing. **Marco Montevecchi:** conceptualization, methodology, supervision, writing – original draft, writing – review and editing. **Cristiano Tomasi:** conceptualization, methodology, writing – review and editing, supervision. **Arianna Giovannini:** conceptualization, methodology, writing – original draft, data curation, investigation, visualization, writing – review and editing. **Nicola Marco Sforza:** conceptualization, methodology, supervision, writing – review and editing. **Luca Landi:** conceptualization, writing – review and editing, supervision, methodology.

## Funding

The authors have nothing to report.

## Disclosure

OSF DOI and date of registration: https://doi.org/10.17605/OSF.IO/MJ36N July 14, 2024.

## Ethics Statement

The authors have nothing to report.

## Consent

The authors have nothing to report.

## Conflicts of Interest

The authors declare no conflicts of interest.

## Supporting information


**Appendix S1:** clr70077‐sup‐0001‐AppendixS1.docx.

**Figure S1:** PRISMA 2020 flow diagram for new systematic reviews which included searches of databases, registers and other sources.

**Table S1:** PRISMA checklist.
**Table S2:** Additional surface roughness parameters reported in included studies (μm, mean ± SD).
**Table S3:** Quality assessment of included studies using the QUIN tool.
**Table S4:** Surface roughness values on modified titanium surfaces (μm, mean ± SD).
**Table S5:** Surface roughness values on machined titanium surfaces (μm, mean ± SD).
**Table S6:** Surface roughness values on zirconia surfaces (μm, mean ± SD).
**Table S7:** Surface roughness values on mixed surfaces (μm, mean ± SD).

## Data Availability

The data that support the findings of this study are available from the corresponding author upon reasonable request.

## References

[clr70077-bib-0002] Azzola, F. , A. C. Ionescu , M. Ottobelli , et al. 2020. “Biofilm Formation on Dental Implant Surface Treated by Implantoplasty: An In Situ Study.” Dentistry Journal 8, no. 2: 40. 10.3390/dj8020040. 32384621 PMC7344745

[clr70077-bib-0003] Batsukh, N. , S. W. Feng , W. F. Lee , et al. 2017. “Effects of *Porphyromonas gingivalis* on Titanium Surface by Different Clinical Treatment.” Journal of Medical and Biological Engineering 37, no. 1: 35–44. 10.1007/s40846-016-0194-0.

[clr70077-bib-0004] Bayrak, M. , N. A. Kocak‐Oztug , K. Gulati , S. Cintan , and E. Cifcibasi . 2022. “Influence of Clinical Decontamination Techniques on the Surface Characteristics of SLA Titanium Implant.” Nanomaterials 12, no. 24: 4481. 10.3390/nano12244481. 36558334 PMC9784882

[clr70077-bib-0005] Berglundh, T. , G. Armitage , M. G. Araujo , et al. 2018. “Peri‐Implant Diseases and Conditions: Consensus Report of Workgroup 4 of the 2017 World Workshop on the Classification of Periodontal and Peri‐Implant Diseases and Conditions.” Journal of Periodontology 89: S313–S318. 10.1002/JPER.17-0739. 29926955

[clr70077-bib-0006] Berglundh, T. , S. Jepsen , B. Stadlinger , and H. Terheyden . 2019. “Peri‐Implantitis and Its Prevention.” Clinical Oral Implants Research 30, no. 2: 150–155. 10.1111/clr.13401. 30636066

[clr70077-bib-0007] Bertoldi, C. , D. Lusuardi , F. Battarra , P. Sassatelli , S. Spinato , and D. Zaffe . 2017. “The Maintenance of Inserted Titanium Implants: In‐Vitro Evaluation of Exposed Surfaces Cleaned With Three Different Instruments.” Clinical Oral Implants Research 28, no. 1: 57–63. 10.1111/clr.12759. 26744027

[clr70077-bib-0008] Biazussi, B. R. , V. Perrotti , C. D'Arcangelo , et al. 2019. “Evaluation of the Effect of Air Polishing With Different Abrasive Powders on the Roughness of Implant Abutment Surface: An In Vitro Study.” Journal of Oral Implantology 45, no. 3: 202–206. 10.1563/AAID-JOI-D-18-00156. 30875272

[clr70077-bib-0009] Bollen, C. M. L. , W. Papaioannou , J. Van Eldere , E. Schepers , M. Quirynen , and D. Van Steenberghe . 1996. “The Influence of Abutment Surface Roughness on Plaque Accumulation and Peri‐Implant Mucositis.” Clinical Oral Implants Research 7, no. 3: 201–211. 10.1034/j.1600-0501.1996.070302.x. 9151584

[clr70077-bib-0010] Cafiero, C. , M. Aglietta , V. Iorio‐Siciliano , G. E. Salvi , A. Blasi , and S. Matarasso . 2017. “Implant Surface Roughness Alterations Induced by Different Prophylactic Procedures: An In Vitro Study.” Clinical Oral Implants Research 28, no. 7: e16–e20. 10.1111/clr.12849. 27283010

[clr70077-bib-0011] Calciolari, E. , N. Mardas , X. Dereka , A. K. Anagnostopoulos , G. T. Tsangaris , and N. Donos . 2018. “Protein Expression During Early Stages of Bone Regeneration Under Hydrophobic and Hydrophilic Titanium Domes. A Pilot Study.” Journal of Periodontal Research 53, no. 2: 174–187. 10.1111/jre.12498. 29063586

[clr70077-bib-0012] Camps‐Font, O. , J. Toledano‐Serrabona , A. Juiz‐Camps , et al. 2023. “Effect of Implantoplasty on Roughness, Fatigue and Corrosion Behavior of Narrow Diameter Dental Implants.” Journal of Functional Biomaterials 14, no. 2: 61. 10.3390/jfb14020061. 36826860 PMC9967762

[clr70077-bib-0013] Chun, K. A. , K. Y. Kum , W. C. Lee , S. H. Baek , H. W. Choi , and W. J. Shon . 2017. “Evaluation of the Safety and Efficiency of Novel Metallic Implant Scaler Tips Manufactured by the Powder Injection Molding Technique.” BMC Oral Health 17, no. 1: 110. 10.1186/s12903-017-0396-z. 28697771 PMC5504980

[clr70077-bib-0014] Costa‐Berenguer, X. , M. García‐García , A. Sánchez‐Torres , M. Sanz‐Alonso , R. Figueiredo , and E. Valmaseda‐Castellón . 2018. “Effect of Implantoplasty on Fracture Resistance and Surface Roughness of Standard Diameter Dental Implants.” Clinical Oral Implants Research 29, no. 1: 46–54. 10.1111/clr.13037. 28736922

[clr70077-bib-0015] Demircioglu, P. , and M. N. Durakbasa . 2011. “Investigations on Machined Metal Surfaces Through the Stylus Type and Optical 3D Instruments and Their Mathematical Modeling With the Help of Statistical Techniques.” Measurements 44, no. 4: 611–619. 10.1016/j.measurement.2010.12.001.

[clr70077-bib-0016] Deng, Y. , X. Liu , A. Xu , et al. 2015. “Effect of Surface Roughness on Osteogenesis In Vitro and Osseointegration In Vivo of Carbon Fiber‐Reinforced Polyetheretherketone–Nanohydroxyapatite Composite.” International Journal of Nanomedicine 10: 1425–1447. 10.2147/IJN.S75557. 25733834 PMC4337592

[clr70077-bib-0017] Derks, J. , and C. Tomasi . 2015. “Peri‐Implant Health and Disease. A Systematic Review of Current Epidemiology.” Journal of Clinical Periodontology 42, no. 16: 158–171. 10.1111/jcpe.12334. 25495683

[clr70077-bib-0018] Dohan Ehrenfest, D. M. , P. G. Coelho , B. S. Kang , Y. T. Sul , and T. Albrektsson . 2010. “Classification of Osseointegrated Implant Surfaces: Materials, Chemistry and Topography.” Trends in Biotechnology 28, no. 4: 198–206. 10.1016/j.tibtech.2009.12.003. 20116873

[clr70077-bib-0019] Duarte, P. M. , A. F. Reis , P. M. de Freitas , and C. Ota‐Tsuzuki . 2009. “Bacterial Adhesion on Smooth and Rough Titanium Surfaces After Treatment With Different Instruments.” Journal of Periodontology 80, no. 11: 1824–1832. 10.1902/jop.2009.090273. 19905952

[clr70077-bib-0020] Durakbasa, M. N. , P. H. Osanna , and P. Demircioglu . 2011. “The Factors Affecting Surface Roughness Measurements of the Machined Flat and Spherical Surface Structures—The Geometry and the Precision of the Surface.” Measurements 44, no. 10: 1986–1999. 10.1016/j.measurement.2011.08.020.

[clr70077-bib-0021] Ercan, E. , T. Arin , L. Kara , C. Çandirli , and C. Uysal . 2014. “Effects of Er,Cr:YSGG Laser Irradiation on the Surface Characteristics of Titanium Discs: An In Vitro Study.” Lasers in Medical Science 29, no. 3: 875–880. 10.1007/s10103-013-1294-5. 23474782

[clr70077-bib-0022] Faccioni, F. , L. Bevilacqua , D. Porrelli , et al. 2021. “Ultrasonic Instrument Effects on Different Implant Surfaces: Profilometry, Energy‐Dispersive X‐Ray Spectroscopy, and Microbiology In Vitro Study.” International Journal of Oral & Maxillofacial Implants 36, no. 3: 520–528. 10.11607/jomi.8140. 34115066

[clr70077-bib-0023] Fakhravar, M. S. , A. Khocht , S. R. Jefferies , and J. B. Suzuki . 2012. “Probing and Scaling Instrumentation on Implant Abutment Surfaces: An In Vitro Study.” Implant Dentistry 21, no. 4: 311–316. 10.1097/ID.0b013e3182588822. 22814556

[clr70077-bib-0024] Gehrke, P. , E. Spanos , C. Fischer , H. Storck , F. Tebbel , and D. Duddeck . 2018. “Influence of Scaling Procedures on the Integrity of Titanium Nitride Coated CAD/CAM Abutments.” Journal of Advanced Prosthodontics 10, no. 3: 197–204. 10.4047/jap.2018.10.3.197. 29930789 PMC6004353

[clr70077-bib-0025] Gennai, S. , J. Bollain , N. Ambrosio , C. Marruganti , F. Graziani , and E. Figuero . 2023. “Efficacy of Adjunctive Measures in Peri‐Implant Mucositis. A Systematic Review and Meta‐Analysis.” Journal of Clinical Periodontology 50, no. 26: 161–187. 10.1111/jcpe.13791. 36792063

[clr70077-bib-0026] Herrera, D. , T. Berglundh , F. Schwarz , et al. 2023. “Prevention and Treatment of Peri‐Implant Diseases—The EFP S3 Level Clinical Practice Guideline.” Journal of Clinical Periodontology 50, no. S26: 4–76. 10.1111/jcpe.13823. 37271498

[clr70077-bib-0027] Huang, Y. S. , C. Y. Hung , and H. H. Huang . 2019. “Surface Changes and Bacterial Adhesion on Implant Abutment Materials After Various Clinical Cleaning Procedures.” Journal of the Chinese Medical Association 82, no. 8: 643–650. 10.1097/JCMA.0000000000000139. 31305347 PMC13048108

[clr70077-bib-0028] Hui, W. L. , D. Ipe , V. Perrotti , et al. 2021. “Novel Technique Using Cold Atmospheric Plasma Coupled With Air‐Polishing for the Treatment of Titanium Discs Grown With Biofilm: An In‐Vitro Study.” Dental Materials 37, no. 2: 359–369. 10.1016/j.dental.2020.11.027. 33358017

[clr70077-bib-0029] Jepsen, S. , T. Berglundh , R. Genco , et al. 2015. “Primary Prevention of Peri‐Implantitis: Managing Peri‐Implant Mucositis.” Journal of Clinical Periodontology 42, no. 16: S152–S157. 10.1111/jcpe.12369. 25626479

[clr70077-bib-0030] Khalil, M. I. , and H. Sakr . 2023. “Implant Surface Topography Following Different Laser Treatments: An In Vitro Study.” Cureus 15, no. 5: e38731. 10.7759/cureus.38731. 37292557 PMC10246922

[clr70077-bib-0031] Kim, H. K. , S. Y. Park , K. Son , et al. 2020. “Alterations in Surface Roughness and Chemical Characteristics of Sandblasted and Acid‐Etched Titanium Implants After Irradiation With Different Diode Lasers.” Applied Sciences (Switzerland) 10, no. 12: 1–10. 10.3390/APP10124167.

[clr70077-bib-0032] Kim, Y. S. , J. B. Park , and Y. Ko . 2019. “Surface Alterations Following Instrumentation With a Nylon or Metal Brush Evaluated With Confocal Microscopy.” Journal of Periodontal and Implant Science 49, no. 5: 310–318. 10.5051/jpis.2019.49.5.310. 31681488 PMC6819692

[clr70077-bib-0033] Kister, F. , O. Specht , M. Warkentin , J. Geis‐Gerstorfer , and F. Rupp . 2017. “Peri‐Implantitis Cleaning Instrumentation Influences the Integrity of Photoactive Nanocoatings.” Dental Materials 33, no. 2: e69–e78. 10.1016/j.dental.2016.10.002. 27832905

[clr70077-bib-0034] Lang, M. , D. Cerutis , T. Miyamoto , and M. Nunn . 2016. “Cell Attachment Following Instrumentation With Titanium and Plastic Instruments, Diode Laser, and Titanium Brush on Titanium, Titanium‐Zirconium, and Zirconia Surfaces.” International Journal of Oral & Maxillofacial Implants 31, no. 4: 799–806. 10.11607/jomi.4440. 27447145

[clr70077-bib-0035] Lee, J. S. , K. Son , S. M. Hwang , et al. 2023. “Effect of Electrocautery and Laser Treatment on the Composition and Morphology of Surface‐Modified Titanium Implants.” Bioengineering 10, no. 11: 1251. 10.3390/bioengineering10111251. 38002374 PMC10669704

[clr70077-bib-0036] Li, R. , L. Wan , X. Zhang , et al. 2022. “Effect of a Neodymium‐Doped Yttrium Aluminium Garnet Laser on the Physicochemical Properties of Contaminated Titanium Surfaces and Macrophage Polarization.” Journal of Periodontal Research 57, no. 3: 533–544. 10.1111/jre.12982. 35266182

[clr70077-bib-0037] Louropoulou, A. , D. E. Slot , and F. Van der Weijden . 2015. “Influence of Mechanical Instruments on the Biocompatibility of Titanium Dental Implants Surfaces: A Systematic Review.” Clinical Oral Implants Research 26, no. 7: 841–850. 10.1111/clr.12365. 24641774

[clr70077-bib-0038] Louropoulou, A. , D. E. Slot , and F. A. Van der Weijden . 2012. “Titanium Surface Alterations Following the Use of Different Mechanical Instruments: A Systematic Review.” Clinical Oral Implants Research 23, no. 6: 643–658. 10.1111/j.1600-0501.2011.02208.x. 21564303

[clr70077-bib-0039] Monje, A. , L. Aranda , K. T. Diaz , et al. 2016. “Impact of Maintenance Therapy for the Prevention of Peri‐Implant Diseases.” Journal of Dental Research 95, no. 4: 372–379. 10.1177/0022034515622432. 26701350

[clr70077-bib-0040] Monje, A. , H. Wang , and J. Nart . 2017. “Association of Preventive Maintenance Therapy Compliance and Peri‐Implant Diseases: A Cross‐Sectional Study.” Journal of Periodontology 88, no. 10: 1030–1041. 10.1902/jop.2017.170135. 28548886

[clr70077-bib-0042] Montevecchi, M. , L. Valeriani , M. F. Salvadori , M. Stefanini , and G. Zucchelli . 2025. “Excess Cement and Peri‐Implant Disease:A Cross‐Sectional clinical endoscopic study.” Journal of Periodontology 96, no. 9: 1–973. 10.1002/JPER.24-0510. PMC12447370 39812473

[clr70077-bib-0043] Page, M. J. , J. E. McKenzie , P. M. Bossuyt , et al. 2021. “The Prisma 2020 Statement: An Updated Guideline for Reporting Systematic Reviews.” BMJ (Clinical Research Edition) 134: 178–189. 10.1136/bmj.n71 PMC8005924 33782057

[clr70077-bib-0044] Park, J. , Y. J. Jang , M. Koh , B. Choi , K. Kim , and Y. Ko . 2013. “In Vitro Analysis of the Efficacy of Ultrasonic Scalers and a Toothbrush for Removing Bacteria From Resorbable Blast Material Titanium Disks.” Journal of Periodontology 84, no. 8: 1191–1198. 10.1902/jop.2012.120369. 23075432

[clr70077-bib-0045] Park, J. B. , Y. Jeon , and Y. Ko . 2015. “Effects of Titanium Brush on Machined and Sand‐Blasted/Acid‐Etched Titanium Disc Using Confocal Microscopy and Contact Profilometry.” Clinical Oral Implants Research 26, no. 2: 130–136. 10.1111/clr.12302. 24299063

[clr70077-bib-0046] Park, J. B. , N. Kim , and Y. Ko . 2012. “Effects of Ultrasonic Scaler Tips and Toothbrush on Titanium Disc Surfaces Evaluated With Confocal Microscopy.” Journal of Craniofacial Surgery 23, no. 5: 1552–1558. 10.1097/SCS.0b013e31825e3ba6. 22976659

[clr70077-bib-0047] Ramel, C. F. , A. Lüssi , M. Özcan , R. E. Jung , C. H. F. Hämmerle , and D. S. Thoma . 2016. “Surface Roughness of Dental Implants and Treatment Time Using Six Different Implantoplasty Procedures.” Clinical Oral Implants Research 27, no. 7: 776–781. 10.1111/clr.12682. 26355907

[clr70077-bib-0048] Renvert, S. , A. M. Roos‐Jansåker , and N. Claffey . 2008. “Non‐Surgical Treatment of Peri‐Implant Mucositis and Peri‐Implantitis: A Literature Review.” Journal of Clinical Periodontology 35, no. SUPPL. 8: 305–315. 10.1111/j.1600-051X.2008.01276.x. 18724858

[clr70077-bib-0049] Rimondini, L. , S. Faré , E. Brambilla , et al. 1997. “The Effect of Surface Roughness on Early In Vivo Plaque Colonization on Titanium.” Journal of Periodontology 68, no. 6: 556–562. 10.1902/jop.1997.68.6.556. 9203099

[clr70077-bib-0050] Sahrmann, P. , S. Winkler , A. Gubler , and T. Attin . 2021. “Assessment of Implant Surface and Instrument Insert Changes due to Instrumentation With Different Tips for Ultrasonic‐Driven Debridement.” BMC Oral Health 21, no. 1: 25. 10.1186/s12903-020-01384-0. 33413296 PMC7791805

[clr70077-bib-0051] Sawase, T. , K. Yoshida , Y. Taira , K. Kamada , M. Atsuta , and K. Baba . 2005. “Abrasion Resistance of Titanium Nitride Coatings Formed on Titanium by Ion‐Beam‐Assisted Deposition.” Journal of Oral Rehabilitation 32, no. 2: 151–157. 10.1111/j.1365-2842.2004.01382.x. 15641983

[clr70077-bib-0052] Sheth, V. H. , N. P. Shah , R. Jain , N. Bhanushali , and V. Bhatnagar . 2024. “Development and Validation of a Risk‐Of‐Bias Tool for Assessing In Vitro Studies Conducted in Dentistry: The QUIN.” Journal of Prosthetic Dentistry 131, no. 6: 1038–1042. 10.1016/j.prosdent.2022.05.019. 35752496

[clr70077-bib-0053] Song, F. , H. Koo , and D. Ren . 2015. “Effects of Material Properties on Bacterial Adhesion and Biofilm Formation.” Journal of Dental Research 94, no. 8: 1027–1034. 10.1177/0022034515587690. 26001706

[clr70077-bib-0054] Stavropoulos, A. , K. Bertl , S. Eren , and K. Gotfredsen . 2019. “Mechanical and Biological Complications After Implantoplasty—A Systematic Review.” Clinical Oral Implants Research 30, no. 9: 833–848. 10.1111/clr.13499. 31254417

[clr70077-bib-0055] Stiesch, M. , J. Grischke , P. Schaefer , and L. J. A. Heitz‐Mayfield . 2023. “Supportive Care for the Prevention of Disease Recurrence/Progression Following Peri‐Implantitis Treatment: A Systematic Review.” Journal of Clinical Periodontology 50, no. 26: 113–134. 10.1111/jcpe.13822. 37339881

[clr70077-bib-0056] Stübinger, S. , F. Homann , C. Etter , M. Miskiewicz , M. Wieland , and R. Sader . 2008. “Effect of Er:YAG,CO_2_ and Diode Laser Irradiation on Surface Properties of Zirconia Endosseous Dental Implants.” Lasers in Surgery and Medicine 40, no. 3: 223–228. 10.1002/lsm.20614. 18366074

[clr70077-bib-0057] Tan, N. C. P. , C. M. Miller , E. Antunes , and D. Sharma . 2022. “Impact of Physical Decontamination Methods on Zirconia Implant Surface and Subsequent Bacterial Adhesion: An In‐Vitro Study.” Clinical and Experimental Dental Research 8, no. 1: 313–321. 10.1002/cre2.486. 34599862 PMC8874065

[clr70077-bib-0058] Taylor, J. B. , A. L. Carrano , and S. G. Kandlikar . 2005. “Characterization of the Effect of Surface Roughness and Texture on Fluid Flow—Past, Present, and Future.” International Journal of Thermal Sciences 45: 11–18. 10.1016/j.ijthermalsci.2006.01.004.

[clr70077-bib-0059] Teughels, W. , N. Van Assche , I. Sliepen , and M. Quirynen . 2006. “Effect of Material Characteristics and/or Surface Topography on Biofilm Development.” Clinical Oral Implants Research 17, no. SUPPL. 2: 68–81. 10.1111/j.1600-0501.2006.01353.x. 16968383

[clr70077-bib-0060] Toma, S. , C. Behets , M. C. Brecx , and J. F. Lasserre . 2018. “In Vitro Comparison of the Efficacy of Peri‐Implantitis Treatments on the Removal and Recolonization of *streptococcus gordonii* Biofilm on Titanium Disks.” Materials 11, no. 12: 2484. 10.3390/ma11122484. 30563297 PMC6316998

[clr70077-bib-0061] Tsampli, A. , S. Rues , H. Kappel , P. Rammelsberg , and S. Kappel . 2024. “In Vitro Pilot Study Comparing a Novel Implantoplasty Sonic Instrumentation Protocol With a Conventional Protocol Using Burs.” Clinical Oral Implants Research 35, no. 3: 340–349. 10.1111/clr.14231. 38225734

[clr70077-bib-0062] Unursaikhan, O. , J. S. Lee , J. K. Cha , et al. 2012. “Comparative Evaluation of Roughness of Titanium Surfaces Treated by Different Hygiene Instruments.” Journal of Periodontal and Implant Science 42, no. 3: 88–94. 10.5051/jpis.2012.42.3.88. 22803010 PMC3395000

[clr70077-bib-0064] Verket, A. , O. C. Koldsland , D. Bunæs , S. A. Lie , and M. Romandini . 2023. “Non‐Surgical Therapy of Peri‐Implant Mucositis—Mechanical/Physical Approaches: A Systematic Review.” Journal of Clinical Periodontology 50, no. S26: 135–145. 10.1111/jcpe.13789. 36802083

[clr70077-bib-0065] Wen, X. , X. Wang , and N. Zhang . 1996. “Microrough Surface of Metallic Biomaterials: A Literature Review.” Bio‐Medical Materials and Engineering 6: 173–189. 8922263

[clr70077-bib-0067] Wennerberg, A. , and T. Albrektsson . 2000. “Suggested Guidelines for the Topographic Evaluation of Implant Surfaces.” International Journal of Oral & Maxillofacial Implants 15: 331–334. 10874798

[clr70077-bib-0066] Wennerberg, A. , and T. Albrektsson . 2010. “On Implant Surfaces: A Review of Current Knowledge and Opinions.” International Journal of Oral & Maxillofacial Implants 25, no. 1: 63–74. 20209188

[clr70077-bib-0068] Ziebolz, D. , G. Schmalz , J. Schmickler , et al. 2017. “Comparison of Different Maintenance Strategies Within Supportive Implant Therapy for Prevention of Peri‐Implant Inflammation During the First Year After Implant Restoration. A Randomized, Dental Hygiene Practice‐Based Multicenter Study.” American Journal of Dentistry 30: 190–196. 29178700

