# Supplementary material for: Impact of Biofilm Decontamination Methods on Implant‐Abutment Surface Integrity: A Systematic Review of Quantitative Studies
Source: Clin Oral Implants Res. 2025 Dec 15;37(3):247–61. doi: 10.1111/clr.70077 (PMC12975692; doi:10.1111/clr.70077)
Supplement: Supplementary file 1 — Appendix S1: clr70077‐sup‐0001‐AppendixS1.docx. [file CLR-37-247-s001.docx]

Search string

**Scopus**

( TITLE-ABS-KEY ( "dental implant*" OR "oral Implant?" OR implantology OR abutment OR "Implant Surface?" ) ) AND ( TITLE-ABS-KEY ( hygien* OR decontamination OR prophylaxis OR maintenance OR debridement OR "non surgical" OR spic OR "Supportive Peri-implant Care" OR "Supportive Care" OR "Supportive Therapy" OR scaling OR scaler OR cavitation OR vibration OR "Biofilm Removal Techniques" OR treatment? OR device? OR clean* OR ultrasonic OR acid OR laser? OR curett* OR polishing OR chemical OR chlorhexidine OR "Air polish*" OR "air abrasion" OR instrument* OR desiccant OR "Rubber cup" OR file OR files OR burs OR bur ) ) AND ( TITLE-ABS-KEY ( alteration? OR rough* OR scratch* OR damage* OR topograph* OR smooth* OR structur* OR texture OR integrity OR surface? OR effect? ) ) AND ( TITLE-ABS-KEY ( profilom* OR prophylom* OR quantitativ* OR ra OR rz OR micron OR µm) )

| No. | **Search terms/combinations** | Results |
| --- | --- | --- |
| #1 | Hygien* OR Decontamination OR Prophylaxis OR Maintenance OR Debridement OR “non surgical” OR SPIC OR “Supportive Peri-implant Care” OR “Supportive Care” OR “Supportive Therapy” OR Scaling OR Scaler OR Cavitation OR Vibration OR “Biofilm Removal Techniques” OR Treatment? OR Device? OR Clean* OR Ultrasonic OR Acid OR Laser? OR Curett* OR Polishing OR Chemical OR Chlorhexidine OR “Air polish*” OR “air abrasion” OR Instrument* OR Desiccant OR “Rubber cup” OR File OR Files OR Burs OR Bur | 19.066.690 |
| #2 | “dental implant*” OR “oral Implant?” OR Implantology OR Abutment OR “Implant Surface?” | 77.228 |
| #3 | Alteration? OR Rough* OR scratch* OR Damage* OR Topograph* OR Smooth* OR Structur* OR Texture OR Integrity OR surface? OR effect? | 24.375.482 |
| #4 | profilom* OR prophylom* OR quantitativ* OR Ra OR Rz OR micron OR µm | 3.637.257 |
|  |  |  |
|  | #1 AND #2 AND #3 AND #4 | 1.173 |
|  |  |  |

**Web of Science**

TS=((Hygien* OR Decontamination OR Prophylaxis OR Maintenance OR Debridement OR Non-surgical OR “non surgical” OR SPIC OR “Supportive Peri-implant Care” OR “Supportive Care” OR “Supportive Therapy” OR Scaling OR Scaler OR Cavitation OR Vibration OR “Biofilm Removal Techniques” OR Treatment* OR Device* OR Clean* OR Ultrasonic OR Acid OR Laser* OR Curett* OR Polishing OR Chemical OR Chlorhexidine OR (Air AND (polish* OR abrasion)) OR Instrument* OR Desiccant OR “Rubber cup” OR File OR Files OR Burs OR Bur) AND (((dent* OR oral) AND Implant*) OR Implantology OR Abutment OR “Implant Surface” OR “implant surfaces”) AND (Alteration* OR Rough* OR scratch* OR Damage* OR Topograph* OR Smooth* OR Structur* OR Texture* OR Integrity OR surface* OR effect*) AND (profilom* OR prophylom* OR quantitativ* OR Ra OR Rz OR micron OR µm))

| No. | **Search terms/combinations** | Results |
| --- | --- | --- |
| #1 | Hygien* OR Decontamination OR Prophylaxis OR Maintenance OR Debridement OR Non-surgical OR “non surgical” OR SPIC OR “Supportive Peri-implant Care” OR “Supportive Care” OR “Supportive Therapy” OR Scaling OR Scaler OR Cavitation OR Vibration OR “Biofilm Removal Techniques” OR Treatment* OR Device* OR Clean* OR Ultrasonic OR Acid OR Laser* OR Curett* OR Polishing OR Chemical OR Chlorhexidine OR (Air AND (polish* OR abrasion)) OR Instrument* OR Desiccant OR “Rubber cup” OR File OR Files OR Burs OR Bur) | 18.603.286 |
| #2 | ((dent* OR oral) AND Implant*) OR Implantology OR Abutment OR “Implant Surface” OR “implant surfaces” | 70.972 |
| #3 | Alteration* OR Rough* OR scratch* OR Damage* OR Topograph* OR Smooth* OR Structur* OR Texture* OR Integrity OR surface* OR effect* | 26.316.346 |
| #4 | profilom* OR prophylom* OR quantitativ* OR Ra OR Rz OR micron OR µm | 1.839.891 |
|  |  |  |
|  | #1 AND #2 AND #3 AND #4 | 1346 |
|  |  |  |

**PubMed**

(Hygien*[Title/Abstract] OR Decontamination[Title/Abstract] OR Prophylaxis[Title/Abstract] OR Maintenance[Title/Abstract] OR Debridement[Title/Abstract] OR Non-surgical[Title/Abstract] OR "non surgical"[Title/Abstract] OR SPIC[Title/Abstract] OR "Supportive Peri-implant Care"[Title/Abstract] OR "Supportive Care"[Title/Abstract] OR "Supportive Therapy"[Title/Abstract] OR Scaling[Title/Abstract] OR Scaler[Title/Abstract] OR Cavitation[Title/Abstract] OR Vibration[Title/Abstract] OR "Biofilm Removal Techniques"[Title/Abstract] OR Treatment*[Title/Abstract] OR Device*[Title/Abstract] OR Clean*[Title/Abstract] OR Ultrasonic[Title/Abstract] OR Acid[Title/Abstract] OR Laser*[Title/Abstract] OR Curett*[Title/Abstract] OR Polishing[Title/Abstract] OR Chemical[Title/Abstract] OR Chlorhexidine[Title/Abstract] OR (Air[Title/Abstract] AND (polish*[Title/Abstract] OR abrasion[Title/Abstract])) OR Instrument*[Title/Abstract] OR Desiccant[Title/Abstract] OR "Rubber cup"[Title/Abstract] OR File[Title/Abstract] OR Files[Title/Abstract] OR Burs[Title/Abstract] OR Bur[Title/Abstract] OR "Decontamination"[Mesh] OR "Preventive Dentistry"[Mesh] OR "Oral Hygiene"[Mesh]) AND (((dent*[Title/Abstract] OR oral[Title/Abstract]) AND Implant*[Title/Abstract]) OR Implantology[Title/Abstract] OR Abutment[Title/Abstract] OR "Implant Surface"[Title/Abstract] OR "implant surfaces"[Title/Abstract] OR dental implants[MeSH Terms] OR dental implantation[MeSH Terms] OR dental implant-abutment design [MeSH Terms]) AND (Alteration*[Title/Abstract] OR Rough*[Title/Abstract] OR scratch*[Title/Abstract] OR Damage*[Title/Abstract] OR Topograph*[Title/Abstract] OR Smooth*[Title/Abstract] OR Structur*[Title/Abstract] OR Texture*[Title/Abstract] OR Integrity[Title/Abstract] OR surface*[Title/Abstract] OR effect*[Title/Abstract]) AND (profilom*[Title/Abstract] OR prophylom*[Title/Abstract] OR quantitativ*[Title/Abstract] OR Ra[Title/Abstract] OR Rz[Title/Abstract] OR micron[Title/Abstract] OR μm[Title/Abstract])

| No. | **Search terms/combinations** | Results |
| --- | --- | --- |
| #1 | (Hygien*[Title/Abstract] OR Decontamination[Title/Abstract] OR Prophylaxis[Title/Abstract] OR Maintenance[Title/Abstract] OR Debridement[Title/Abstract] OR Non-surgical[Title/Abstract] OR "non surgical"[Title/Abstract] OR SPIC[Title/Abstract] OR "Supportive Peri-implant Care"[Title/Abstract] OR "Supportive Care"[Title/Abstract] OR "Supportive Therapy"[Title/Abstract] OR Scaling[Title/Abstract] OR Scaler[Title/Abstract] OR Cavitation[Title/Abstract] OR Vibration[Title/Abstract] OR "Biofilm Removal Techniques"[Title/Abstract] OR Treatment*[Title/Abstract] OR Device*[Title/Abstract] OR Clean*[Title/Abstract] OR Ultrasonic[Title/Abstract] OR Acid[Title/Abstract] OR Laser*[Title/Abstract] OR Curett*[Title/Abstract] OR Polishing[Title/Abstract] OR Chemical[Title/Abstract] OR Chlorhexidine[Title/Abstract] OR (Air[Title/Abstract] AND (polish*[Title/Abstract] OR abrasion[Title/Abstract])) OR Instrument*[Title/Abstract] OR Desiccant[Title/Abstract] OR "Rubber cup"[Title/Abstract] OR File[Title/Abstract] OR Files[Title/Abstract] OR Burs[Title/Abstract] OR Bur[Title/Abstract] | 9.512.024 |
| #2 | "Decontamination"[Mesh] OR "Preventive Dentistry"[Mesh] OR "Oral Hygiene"[Mesh] | 43.416 |
| #3 | ((dent*[Title/Abstract] OR oral[Title/Abstract]) AND Implant*[Title/Abstract]) OR Implantology[Title/Abstract] OR Abutment[Title/Abstract] OR "Implant Surface"[Title/Abstract] OR "implant surfaces"[Title/Abstract] | 58.837 |
| #4 | dental implants[MeSH Terms] OR dental implantation[MeSH Terms] OR dental implant-abutment design [MeSH Terms] | 42.310 |
| #5 | Alteration*[Title/Abstract] OR Rough*[Title/Abstract] OR scratch*[Title/Abstract] OR Damage*[Title/Abstract] OR Topograph*[Title/Abstract] OR Smooth*[Title/Abstract] OR Structur*[Title/Abstract] OR Texture*[Title/Abstract] OR Integrity[Title/Abstract] OR surface*[Title/Abstract] OR effect*[Title/Abstract] | 12.637206 |
| #6 | profilom*[Title/Abstract] OR prophylom*[Title/Abstract] OR quantitativ*[Title/Abstract] OR Ra[Title/Abstract] OR Rz[Title/Abstract] OR micron[Title/Abstract] OR μm[Title/Abstract]) | 1.221.421 |
|  |  |  |
|  | (#1 OR #2) AND (#3 OR #4) AND #5 AND #6 | 1289 |
