# Supplementary material for: Impact of Biofilm Decontamination Methods on Implant‐Abutment Surface Integrity: A Systematic Review of Quantitative Studies
Source: Clin Oral Implants Res. 2025 Dec 15;37(3):247–61. doi: 10.1111/clr.70077 (PMC12975692; doi:10.1111/clr.70077)
Supplement: Supplementary file 2 — Figure S1: PRISMA 2020 flow diagram for new systematic reviews which included searches of databases, registers and other sources. [file CLR-37-247-s002.docx]

Figure 1: PRISMA 2020 flow diagram for new systematic reviews which included searches of databases, registers and other sources

**Identification of studies via other methods**

**Identification of studies via databases and registers**

Records identified from:

Citation searching (n=9)

Records removed *before screening*:

Duplicate records removed (n=1463)

Records identified from:

Scopus (n=1173)

Web of Science (n=1346)

Medline via PubMed (n=1289)

**Identification**

Records excluded

(n=2258)

Records screened

(n=2345)

Reports sought for retrieval

(n=9)

Reports not retrieved (n=16):

Full text not retrieved (n =8)

Data not retrieved (n=8)

Reports not retrieved

(n=1)

**Screening**

Reports sought for retrieval

(n=87)

Reports excluded (n=6):

Threads zone (n=1)

Prosthetic zone (n=4)

Peri-implantitis treatment (n=1)

Reports excluded (n=44):

Wrong outcome (n=10)

Threads zone (n=9)

Peri-implantitis treatment (n=2)

No control group (n=4)

No quantitative analysis (n=14)

No surface analysis (n=4)

No materials and methods (n=1)

Wrong language (n=1)

Wrong parameters (n=3)

Wrong measure (n=3)

Reports assessed for eligibility (n=8)

Reports assessed for eligibility (n=71)

Studies included in review

(n=29)

**Included**
