# Supplementary material for: Impact of Biofilm Decontamination Methods on Implant‐Abutment Surface Integrity: A Systematic Review of Quantitative Studies
Source: Clin Oral Implants Res. 2025 Dec 15;37(3):247–61. doi: 10.1111/clr.70077 (PMC12975692; doi:10.1111/clr.70077)
Supplement: Supplementary file 3 — Table S1: PRISMA checklist. Table S2: Additional surface roughness parameters reported in included studies (μm, mean ± SD). Table S3: Quality assessment of included studies using the QUIN tool. Table S4: Surface roughness values on modified titanium surfaces (μm, mean ± SD). Table S5: Surface roughness values on machined titanium surfaces (μm, mean ± SD). Table S6: Surface roughness values on zirconia surfaces (μm, mean ± SD). Table S7: Surface roughness values on mixed surfaces (μm, mean ± SD). [file CLR-37-247-s003.zip › clr70077-sup-0003-TableS2.docx]

Table S2: Additional surface roughness parameters reported in included studies (µm; mean ± SD)

| Material | Surface | Instrument | Type | Article | Measuring instrument | Parameter | Control | Test |
| --- | --- | --- | --- | --- | --- | --- | --- | --- |
| Ti | iniciell | Ultrasonic | Carbon | Sahrmann, 2021 | profilometer | Rt | 8,60 ± 0,93 | 6,25 ± 1,09 |
| Ti | iniciell | Ultrasonic | Metal | Sahrmann, 2021 | profilometer | Rt | 8,60 ± 0,90 | 4,30 ± 0,90 |
| Ti | iniciell | Ultrasonic | Plastic | Sahrmann, 2021 | profilometer | Rt | 8,60 ± 0,91 | 5,42 ± 0,91 |
| Ti | iniciell | Ultrasonic | Resin | Sahrmann, 2021 | profilometer | Rt | 8,60 ± 0,94 | 8,27 ± 1,03 |
| Ti | iniciell | Ultrasonic | Titanium | Sahrmann, 2021 | profilometer | Rt | 8,60 ± 0,92 | 5,95 ± 1,43 |
| Ti | machined | Brush | Metal | Kim, 2019 | microscope | Rsk | 0,76±0,39 | 0,79±0,72 |
| Ti | machined | Brush | Metal | Kim, 2019 | microscope | Sdr | 6,53±4,55 | 14,18±4,12 |
| Ti | machined | Brush | Metal | Kim, 2019 | microscope | Sku | 5,93±1,86 | 6,26±4,15 |
| Ti | machined | Brush | Metal | Kim, 2019 | microscope | Ssk | 0,78±0,37 | 0,77±0,38 |
| Ti | machined | Brush | Metal | Kim, 2019 | microscope | Sz | 2,47±0,67 | 7,0±0,65 |
| Ti | machined | Brush | Nylon | Sawase, 2005 | profilometer | Rmax | 0,131 | 0,848 |
| Ti | machined | Brush | Nylon | Kim, 2019 | microscope | Rsk | 0,76±0,38 | 0,86±0,81 |
| Ti | machined | Brush | Nylon | Kim, 2019 | microscope | Sdr | 6,53±4,54 | 20,82±16,84 |
| Ti | machined | Brush | Nylon | Kim, 2019 | microscope | Sku | 5,93±1,85 | 10,69±21,43 |
| Ti | machined | Brush | Nylon | Kim, 2019 | microscope | Ssk | 0,78±0,36 | 0,73±0,65 |
| Ti | machined | Brush | Nylon | Kim, 2019 | microscope | Sz | 2,47±0,66 | 4,52±0,88 |
| Ti | machined | Brush | Titanium | Park, 2013B | profilometer | Rsk | 0,71±0,74 | 0,78±0,72 |
| Ti | machined | Brush | Titanium | Park, 2013B | profilometer | Ssk | 0,66±0,68 | 0,94±0,531 |
| Ti | machined | Brush | Titanium | Park, 2013B | profilometer | Sz | 4,33±1,77 | 3,57±1,02 |
| Ti | machined | Curette | Metal | Bertoldi, 2016 | profilometer | Rt | range 0,65-2,1 | range 0,6-1,8 |
| Ti | machined | Curette | Titanium | Bertoldi, 2016 | profilometer | Rt | range0,6-1,8 | range 0,15-0,7 |
| Ti | machined | Scaler | Metal | Sawase, 2005 | profilometer | Rmax | 0,131 | 3,1 |
| Ti | machined | Scaler | Plastic | Sawase, 2005 | profilometer | Rmax | 0,131 | 0,725 |
| Ti | machined | Toothbrush |  | Sawase, 2005 | profilometer | Rmax | 0,131 | 0,702 |
| Ti | machined | Toothbrush |  | Park, 2013A | microscope | Ssk | 0,62±0,30 | 0,52±0,28 |
| Ti | machined | Toothbrush |  | Park, 2013A | microscope | Sz | 19,12±1,20 | 19,19±1,75 |
| Ti | machined | Ultrasonic | Bronze | Chun, 2017 | microscope | Rmax | 3,4 | 4,2 |
| Ti | machined | Ultrasonic | Carbon | Sahrmann, 2021 | profilometer | Rt | 3,10 ± 0,60 | 2,55 ± 0,46 |
| Ti | machined | Ultrasonic | Carbon | Sahrmann, 2021 | profilometer | Rt | 0,26 ± 0,11 | 0,53 ± 0,30 |
| Ti | machined | Ultrasonic | Carbon | Park, 2013A | microscope | Ssk | 0,62±0,29 | 0,44±0,37 |
| Ti | machined | Ultrasonic | Carbon | Park, 2013A | microscope | Sz | 19,12±1,19 | 18,85±1,40 |
| Ti | machined | Ultrasonic | Copper | Chun, 2017 | microscope | Rmax | 3,4 | 4,1 |
| Ti | machined | Ultrasonic | Metal | Chun, 2017 | microscope | Rmax | 3,4 | 10 |
| Ti | machined | Ultrasonic | Metal | Chun, 2017 | microscope | Rmax | 3,4 | 119,3 |
| Ti | machined | Ultrasonic | Metal | Sahrmann, 2021 | profilometer | Rt | 3,10 ± 0,57 | 3,47 ± 0,69 |
| Ti | machined | Ultrasonic | Metal | Sahrmann, 2021 | profilometer | Rt | 0,26 ± 0,08 | 2,87 ± 0,61 |
| Ti | machined | Ultrasonic | Metal | Park, 2013A | microscope | Ssk | 0,62±0,26 | 1,11±0,46 |
| Ti | machined | Ultrasonic | Metal | Park, 2013A | microscope | Ssk | 0,62±0,28 | 0,75±0,22 |
| Ti | machined | Ultrasonic | Metal | Park, 2013A | microscope | Sz | 19,12±1,16 | 15,31±1,30 |
| Ti | machined | Ultrasonic | Metal | Park, 2013A | microscope | Sz | 19,12±1,18 | 18,24±1,33 |
| Ti | machined | Ultrasonic | Plastic | Bertoldi, 2016 | profilometer | Rt | range 0,5-2 | range 0,2-0,65 |
| Ti | machined | Ultrasonic | Plastic | Sahrmann, 2021 | profilometer | Rt | 3,10 ± 0,58 | 2,78 ± 0,65 |
| Ti | machined | Ultrasonic | Plastic | Sahrmann, 2021 | profilometer | Rt | 0,26 ± 0,09 | 0,45 ± 0,18 |
| Ti | machined | Ultrasonic | Plastic | Park, 2013A | microscope | Ssk | 0,62±0,27 | 0,75±0,26 |
| Ti | machined | Ultrasonic | Plastic | Park, 2013A | microscope | Sz | 19,12±1,17 | 18,72±1,35 |
| Ti | machined | Ultrasonic | Resin | Sahrmann, 2021 | profilometer | Rt | 3,10 ± 0,61 | 2,84 ± 0,53 |
| Ti | machined | Ultrasonic | Resin | Sahrmann, 2021 | profilometer | Rt | 0,26 ± 0,12 | 0,40 ± 0,15 |
| Ti | machined | Ultrasonic | Titanium | Sahrmann, 2021 | profilometer | Rt | 3,10 ± 0,59 | 4,44 ± 1,31 |
| Ti | machined | Ultrasonic | Titanium | Sahrmann, 2021 | profilometer | Rt | 0,26 ± 0,10 | 3,51 ± 1,75 |
| Ti | machined | Ultrasonic |  | Sawase, 2005 | profilometer | Rmax | 0,131 | 8,977 |
| Ti | modified | Brush | Metal | Kim, 2019 | microscope | Rsk | 0,26±0,26 | 0,69±0,33 |
| Ti | modified | Brush | Metal | Kim, 2019 | microscope | Sdr | 88,50±3,93 | 66,50±5,07 |
| Ti | modified | Brush | Metal | Kim, 2019 | microscope | Sku | 3,83±0,68 | 3,68±0,64 |
| Ti | modified | Brush | Metal | Kim, 2019 | microscope | Ssk | 0,47±0,23 | 0,77±0,38 |
| Ti | modified | Brush | Metal | Kim, 2019 | microscope | Sz | 13,88±1,06 | 11,66±0,57 |
| Ti | modified | Brush | Nylon | Kim, 2019 | microscope | Rsk | 0,26±0,25 | 0,21±0,26 |
| Ti | modified | Brush | Nylon | Kim, 2019 | microscope | Sdr | 88,50±3,92 | 83,01±6,58 |
| Ti | modified | Brush | Nylon | Kim, 2019 | microscope | Sku | 3,83±0,68 | 3,42±0,81 |
| Ti | modified | Brush | Nylon | Kim, 2019 | microscope | Ssk | 0,47±0,22 | 0,73±0,65 |
| Ti | modified | Brush | Nylon | Kim, 2019 | microscope | Sz | 13,88±1,05 | 13,12±0,55 |
| Ti | modified | Brush | Titanium | Bayark, 2022 | profilometer | Rq | 2,84 ± 0,18 | 2,06 ± 0,09 |
| Ti | modified | Brush | Titanium | Park, 2013B | profilometer | Rsk | -0,21±0,57 | 0,33±0,39 |
| Ti | modified | Brush | Titanium | Bayark, 2022 | profilometer | Rt | 24,39 ± 5,88 | 16,68 ± 1,69 |
| Ti | modified | Brush | Titanium | Park, 2013B | profilometer | Ssk | 0,10±0,13 | 0,43±0,41 |
| Ti | modified | Brush | Titanium | Park, 2013B | profilometer | Sz | 13,52±0,76 | 13,26±0,66 |
| Ti | modified | Curette | Titanium | Bayark, 2022 | profilometer | Rq | 2,84 ± 0,17 | 1,58 ± 0,24 |
| Ti | modified | Curette | Titanium | Bayark, 2022 | profilometer | Rt | 24,39 ± 5,87 | 18,13 ± 4,95 |
| Ti | modified | Curette | Titanium | Gehrke, 2018 | profilometer | Sdr | 0,85 ± 0,14 | 0,46 ± 0,11 |
| Ti | modified | Laser | Diode | Bayark, 2022 | profilometer | Rq | 2,84 ± 0,19 | 2,75 ± 0,31 |
| Ti | modified | Laser | Diode | Bayark, 2022 | profilometer | Rt | 24,39 ± 5,89 | 32,23 ± 7,57 |
| Ti | modified | Laser | Diode630 | Khalil, 2023 | profilometer | Rq | 3,57±0,20 | 4,35±0,26 |
| Ti | modified | Laser | Diode808 | Khalil, 2023 | profilometer | Rq | 3,57±0,21 | 4,72±0,56 |
| Ti | modified | Laser | Er,Cr:YSGG | Khalil, 2023 | profilometer | Rq | 3,57±0,19 | 4,49±0,34 |
| Ti | modified | Scaler | Acrylic | Gehrke, 2018 | profilometer | Sdr | 0,85 ± 0,14 | 0,79 ± 0,10 |
| Ti | modified | Ultrasonic | Carbon | Sahrmann, 2021 | profilometer | Rt | 9,01 ± 1,43 | 6,75 ± 1,14 |
| Ti | modified | Ultrasonic | Metal | Sahrmann, 2021 | profilometer | Rt | 9,01 ± 1,40 | 4,33 ± 1,12 |
| Ti | modified | Ultrasonic | Metal | Gehrke, 2018 | profilometer | Sdr | 0,85 ± 0,14 | 0,57 ± 0,23 |
| Ti | modified | Ultrasonic | Plastic | Sahrmann, 2021 | profilometer | Rt | 9,01 ± 1,41 | 6,05 ± 1,33 |
| Ti | modified | Ultrasonic | Resin | Sahrmann, 2021 | profilometer | Rt | 9,01 ± 1,44 | 8,87 ± 2,11 |
| Ti | modified | Ultrasonic | Titanium | Sahrmann, 2021 | profilometer | Rt | 9,01 ± 1,42 | 5,24 ± 1,48 |
| Ti | modified-rbm | Brush | Metal | Kim, 2019 | microscope | Rsk | 0,25±0,39 | 0,52±0,41 |
| Ti | modified-rbm | Brush | Metal | Kim, 2019 | microscope | Sdr | 106,09±6,72 | 83,79±8,82 |
| Ti | modified-rbm | Brush | Metal | Kim, 2019 | microscope | Sku | 4,54±0,57 | 4,72±0,99 |
| Ti | modified-rbm | Brush | Metal | Kim, 2019 | microscope | Ssk | 0,61±0,24 | 0,84±0,28 |
| Ti | modified-rbm | Brush | Metal | Kim, 2019 | microscope | Sz | 9,58±1,08 | 17,98±1,29 |
| Ti | modified-rbm | Brush | Nylon | Kim, 2019 | microscope | Rsk | 0,25±0,38 | 0,26±0,98 |
| Ti | modified-rbm | Brush | Nylon | Kim, 2019 | microscope | Sdr | 106,09±6,71 | 107,00±25,27 |
| Ti | modified-rbm | Brush | Nylon | Kim, 2019 | microscope | Sku | 4,54±0,56 | 4,36±0,90 |
| Ti | modified-rbm | Brush | Nylon | Kim, 2019 | microscope | Ssk | 0,61±0,23 | 0,62±0,24 |
| Ti | modified-rbm | Brush | Nylon | Kim, 2019 | microscope | Sz | 9,58±1,07 | 19,33±1,47 |
| TiN | machined | Brush | Nylon | Sawase, 2005, 2012 | profilometer | Rmax | 0,183 | 0,192 |
| TiN | machined | Scaler | Metal | Sawase, 2005 | profilometer | Rmax | 0,183 | 1,347 |
| TiN | machined | Scaler | Plastic | Sawase, 2005 | profilometer | Rmax | 0,183 | 0,184 |
| TiN | machined | Toothbrush |  | Sawase, 2005 | profilometer | Rmax | 0,183 | 0,206 |
| TiN | machined | Ultrasonic |  | Sawase, 2005 | profilometer | Rmax | 0,183 | 2,539 |
| Zr | machined | Laser | CO2 | Stübinger, 2008 | microscope | St | 0,2633±0,0873 | 2,5±1,5451 |
| Zr | machined | Laser | CO2 | Stübinger, 2008 | microscope | St | 0,2633±0,0873 | 5,6833±0,9134 |
| Zr | machined | Laser | CO2 | Stübinger, 2008 | microscope | St | 0,2633±0,0873 | 3,2567±2,9787 |
| Zr | machined | Laser | CO2 | Stübinger, 2008 | microscope | St | 0,2633±0,0873 | 3,225±1,3964 |
| Zr | machined | Laser | CO2 | Stübinger, 2008 | microscope | St | 0,2633±0,0873 | 8,1517±0,7591 |
| Zr | modified | Air-abrasive | glycine | Tan, 2021 | microscope | Sku | 3,82 | 5,54 |
| Zr | modified | Air-abrasive | glycine | Tan, 2021 | microscope | Ssk | -0,08 | -0,02 |
| Zr | modified | Air-abrasive | glycine | Tan, 2021 | microscope | Sz | 56,73 | 54,45 |
| Zr | modified | Curette | Plastic | Tan, 2021 | microscope | Sku | 3,82 | 3,58 |
| Zr | modified | Curette | Plastic | Tan, 2021 | microscope | Ssk | -0,08 | 0,03 |
| Zr | modified | Curette | Plastic | Tan, 2021 | microscope | Sz | 56,73 | 50,15 |
| Zr | modified | Curette | Titanium | Tan, 2021 | microscope | Sku | 3,82 | 10,06 |
| Zr | modified | Curette | Titanium | Tan, 2021 | microscope | Ssk | -0,08 | 0,94 |
| Zr | modified | Curette | Titanium | Tan, 2021 | microscope | Sz | 56,73 | 57,42 |
| Zr | modified | Ultrasonic |  | Tan, 2021 | microscope | Sku | 3,82 | 18,62 |
| Zr | modified | Ultrasonic |  | Tan, 2021 | microscope | Ssk | -0,08 | 1,74 |
| Zr | modified | Ultrasonic |  | Tan, 2021 | microscope | Sz | 56,73 |  |
