# Supplementary material for: Impact of Biofilm Decontamination Methods on Implant‐Abutment Surface Integrity: A Systematic Review of Quantitative Studies
Source: Clin Oral Implants Res. 2025 Dec 15;37(3):247–61. doi: 10.1111/clr.70077 (PMC12975692; doi:10.1111/clr.70077)
Supplement: Supplementary file 3 — Table S1: PRISMA checklist. Table S2: Additional surface roughness parameters reported in included studies (μm, mean ± SD). Table S3: Quality assessment of included studies using the QUIN tool. Table S4: Surface roughness values on modified titanium surfaces (μm, mean ± SD). Table S5: Surface roughness values on machined titanium surfaces (μm, mean ± SD). Table S6: Surface roughness values on zirconia surfaces (μm, mean ± SD). Table S7: Surface roughness values on mixed surfaces (μm, mean ± SD). [file CLR-37-247-s003.zip › clr70077-sup-0005-TableS3@Supplementary Table 3.docx]

Supplementary Table 3: Quality assessment of included studies using the QUIN tool

| **Articles** | **Clearly stated aims/**  **objective** | **Detailed explanation of sample size calculation** | **Detailed explanation of sampling technique** | **Details of comparison group** | **Detailed explanation of methodology** | **Operator details** | **Randomization** | **Method of measurement of outcome** | **Outcome assessor details** | **Blinding** | **Statistical analysis** | **Presentation of results** | **Risk of bias** |
| --- | --- | --- | --- | --- | --- | --- | --- | --- | --- | --- | --- | --- | --- |
| Batsukh, 2017 | 2 | 0 | 2 | 2 | 1 | 0 | 2 | 1 | 0 | 0 | 2 | 2 | Medium |
| Bayark, 2022 | 2 | 1 | 2 | 2 | 1 | 2 | 0 | 2 | 0 | 0 | 2 | 2 | Medium |
| Bertoldi, 2016 | 2 | 0 | 1 | 2 | 2 | 2 | 2 | 1 | 1 | 0 | 0 | 2 | Medium |
| Biazussi, 2019 | 2 | 2 | 2 | 2 | 1 | 0 | 2 | 1 | 0 | 0 | 1 | 2 | Medium |
| Cafiero, 2016 | 2 | 1 | 1 | 2 | 1 | 1 | 2 | 2 | 0 | 0 | 1 | 2 | Medium |
| Chun, 2017 | 2 | 1 | 2 | 2 | 2 | 1 | 0 | 2 | 0 | 0 | 2 | 2 | Medium |
| Duarte, 2009 | 2 | 1 | 2 | 2 | 1 | 1 | 2 | 2 | 0 | 0 | 2 | 2 | Low |
| Ercan, 2013 | 2 | 0 | 2 | 2 | 1 | 0 | 0 | 2 | 0 | 0 | 0 | 2 | High |
| Faccioni, 2021 | 2 | 0 | 2 | 1 | 1 | 2 | 0 | 2 | 0 | 0 | 2 | 2 | Medium |
| Fakhravar, 2012 | 2 | 0 | 1 | 1 | 1 | 1 | 2 | 2 | 0 | 2 | 2 | 2 | Medium |
| Gehrke, 2018 | 2 | 1 | 1 | 2 | 2 | 0 | 2 | 2 | 0 | 0 | 2 | 2 | Medium |
| Huang, 2019 | 2 | 1 | 2 | 2 | 2 | 2 | 2 | 2 | 0 | 0 | 2 | 2 | Low |
| Hui, 2021 | 2 | 1 | 2 | 2 | 1 | 2 | 0 | 2 | 0 | 0 | 1 | 1 | Medium |
| Khalil, 2023 | 1 | 1 | 1 | 2 | 1 | 1 | 2 | 1 | 0 | 2 | 1 | 2 | Medium |
| Kim, 2019 | 2 | 1 | 1 | 2 | 1 | 1 | 2 | 2 | 0 | 0 | 1 | 2 | Medium |
| Kim, 2020 | 2 | 1 | 1 | 2 | 1 | 1 | 0 | 0 | 0 | 0 | 1 | 2 | High |
| Kister, 2017 | 2 | 1 | 2 | 2 | 1 | 0 | 0 | 2 | 0 | 0 | 2 | 2 | Medium |
| Lang, 2016 | 2 | 2 | 2 | 2 | 1 | 1 | 0 | 2 | 1 | 0 | 2 | 2 | Low |
| Lee, 2023 | 2 | 0 | 2 | 2 | 2 | 2 | 0 | 2 | 0 | 0 | 2 | 2 | Medium |
| Li, 2022 | 2 | 0 | 2 | 2 | 1 | 0 | 2 | 1 | 0 | 0 | 1 | 2 | Medium |
| Park, 2012 | 2 | 0 | 2 | 2 | 2 | 1 | 0 | 2 | 0 | 0 | 1 | 2 | Medium |
| Park, 2013 | 2 | 1 | 1 | 2 | 1 | 1 | 2 | 2 | 0 | 0 | 1 | 2 | Medium |
| Park, 2015 | 2 | 0 | 1 | 2 | 2 | 1 | 2 | 2 | 0 | 0 | 2 | 2 | Medium |
| Sahrmann, 2021 | 2 | 1 | 2 | 1 | 2 | 0 | 0 | 2 | 0 | 0 | 2 | 2 | Medium |
| Sawase, 2005 | 1 | 0 | 2 | 1 | 2 | 2 | 2 | 2 | 0 | 0 | 2 | 2 | Medium |
| Stübinger, 2008 | 2 | 0 | 2 | 2 | 1 | 0 | 0 | 1 | 0 | 0 | 1 | 2 | High |
| Tan, 2021 | 2 | 0 | 2 | 2 | 1 | 0 | 2 | 2 | 0 | 0 | 2 | 2 | Medium |
| Toma, 2018 | 2 | 1 | 1 | 2 | 1 | 0 | 0 | 2 | 0 | 0 | 2 | 2 | Medium |
| Unursaikhan, 2021 | 2 | 1 | 1 | 1 | 1 | 1 | 0 | 2 | 0 | 0 | 1 | 2 | Medium |

QUIN score: 2 = adequately specified; 1 = inadequately specified; 0 = not specified
