# Supplementary material for: Impact of Biofilm Decontamination Methods on Implant‐Abutment Surface Integrity: A Systematic Review of Quantitative Studies
Source: Clin Oral Implants Res. 2025 Dec 15;37(3):247–61. doi: 10.1111/clr.70077 (PMC12975692; doi:10.1111/clr.70077)
Supplement: Supplementary file 3 — Table S1: PRISMA checklist. Table S2: Additional surface roughness parameters reported in included studies (μm, mean ± SD). Table S3: Quality assessment of included studies using the QUIN tool. Table S4: Surface roughness values on modified titanium surfaces (μm, mean ± SD). Table S5: Surface roughness values on machined titanium surfaces (μm, mean ± SD). Table S6: Surface roughness values on zirconia surfaces (μm, mean ± SD). Table S7: Surface roughness values on mixed surfaces (μm, mean ± SD). [file CLR-37-247-s003.zip › clr70077-sup-0006-TableS4-S7@Supplementary Table 4-7.docx]

Supplementary Table 4. Surface roughness values on modified titanium surfaces (µm. mean ± SD)

| **Instrument** | **Type** | **Article** | **Ra** | **Sa** | **Rz** | **Protocol/Surface** | **Measurement** |
| --- | --- | --- | --- | --- | --- | --- | --- |
| Air-Polishing | Erythritol 14μm | Hui. 2021 | NA | **T**: 3.225  **C**: 2.355  **𝛿**: 0.87 (37%) | NA | 20s. distance 10mm. Sandblasted surface | Profilometer |
|  | Glycine 25μm | Toma. 2018 | **T**: 1.31±0.14  **C**: 1.65±0.107  **𝛿**: -0.34 (-21%) | NA | **T**: 9.22±0.27  **C**: 9.79±0.34  **𝛿**: -0.57 (-6%) | 30s. non-contact mode. Sandblasted surface | Profilometer |
|  | SodiumBicarbonate | Duarte. 2009 | **T**: 0.69±0.008  **C**: 0.70±0.007  **𝛿**: -0.01 (-1%) | NA | NA | Medium power. distance 5mm. SLA surface | Profilometer |
| Brush | Metal | Kim. 2019 | **T**: 1.79±0.31* **C**: 2.18±0.47  **𝛿**: -0.39 (-18%) | **T**: 1.88±0.12** **C**: 2.21±0.29  **𝛿**: -0.33 (-15%) | **T**: 5.85±0.62** **C**: 6.71±0.96  **𝛿**: -0.86 (-13%) | 40s. 1.000rpm. pressure ≃10g. SLA surface | Microscope |
|  | Metal | Kim. 2019 | **T**: 2.58±0.59  **C**: 2.75±0.51  **𝛿**: -0.17 (-6%) | **T**: 2.77±0.43 **C**: 2.81±0.12  **𝛿**: -0.05 (-2%) | **T**: 6.77±1.63*  **C**: 8.32±1.61  **𝛿**: -1.55 (-19%) | 40s. 1.000rpm. pressure ≃10g. RBM surface | Microscope |
|  | Nylon | Kim. 2019 | **T**: 1.87±0.23*  **C**: 2.18±0.47  **𝛿**: -0.31 (-14%) | **T**: 1.94±0.10** **C**: 2.21±0.29  **𝛿**: -0.27 (-12%) | **T**: 6.52±0.64 **C**: 6.71±0.96  **𝛿**: -0.19 (-3%) | 40s. 1.000rpm. pressure ≃10g. SLA surface | Microscope |
|  | Nylon | Kim. 2019 | **T**: 2.68±0.57  **C**: 2.75±0.51  **𝛿**: -0.07 (-3%) | **T**: 2.94±0.32 **C**: 2.81±0.12  **𝛿**: 0.13 (5%) | **T**: 7.58±1.20*  **C**: 8.32±1.61  **𝛿**: -0.74 (-9%) | 40s. 1.000rpm. pressure ≃10g. RBM surface | Microscope |
|  | Titanium | Bayrak. 2022 | **T**: 1.62±0.05  **C**: 2.25±0.14  **𝛿**: -0.63 (-28%) | NA | NA | 60s. 920rpm. SLA surface | Profilometer |
|  | Titanium | Park. 2015 | **T**: 1.73±0.20  **C**: 1.87±0.21  **𝛿**: -0.14 (-7%) | **T**: 1.89±0.10 **C**: 1.94±0.09  **𝛿**: -0.05 (-3%) | **T**: 6.35±0.66* **C**: 7.25±0.63  **𝛿**: -0.9 (-12%) | 40s. 300rpm. SLA surface | Microscope |
|  | Titanium | Park. 2015 | **T**: 1.40±0.07  **C**: 1.36±0.11  **𝛿**: 0.04 (3%) | NA | NA | 40s. 300rpm. SLA surface | Profilometer |
|  | Titanium | Toma. 2018 | **T**: 1.22±0.31  **C**: 1.65±0.107  **𝛿**: -0.43 (26%) | NA | **T**: 8.76±0.15  **C**: 9.79±0.34  **𝛿**: -1.03(-11%) | 30s. low speed. 900osc/min. SandBlasted surface | Profilometer |
| Cold Atmospheric Plasma |  | Hui. 2021 | NA | **T**: 2.8275 **C**: 2.355  **𝛿**: 0.47 (20%) | NA | Distance 5mm. 1.4Hz. 10kV. 2°C | Profilometer |
| Curette | Metal | Duarte. 2009 | **T**: 0.73±0.27  **C**: 0.71±0.03  **𝛿**: 0.02 (3%) | NA | NA | 30strokes. 70°. SLA surface | Profilometer |
|  | Plastic | Duarte. 2009 | **T**: 0.70±0.08  **C**: 0.70±0.03  **𝛿**: 0 (0%) | NA | NA | 30strokes. 70°. SLA surface | Profilometer |
|  | Plastic | Toma. 2018 | **T**: 1.61±0.17  **C**: 1.65±0.107  **𝛿**: -0.04 (-2%) | NA | **T**: 9.98±0.24 **C**: 9.79±0.34  **𝛿**: 0.19 (2%) | 30s. 70°. SandBlasted surface | Profilometer |
|  | Titanium | Bayrak. 2022 | **T**: 1.22±0.18  **C**: 2.25±0.14  **𝛿**: -1.03 (-46%) | NA | NA | 60s. 45°. SLA surface | Profilometer |
| Laser | 650Diode | Khalil. 2023 | **T**: 3.45±0.19*+  **C**: 2.81±0.10  **𝛿**: 0.64 (23%) | NA | NA | 50mW. 60mJ. SLA surface | Profilometer |
|  | 808Diode | Khalil. 2023 | **T**: 3.77±0.42**  **C**: 2.81±0.10  **𝛿**: 0.96 (34%) | NA | NA | 1W. 120mJ. SLA surface | Profilometer |
|  | Diode | Bayark. 2022 | **T**: 2.08±0.14  **C**: 2.25±0.14  **𝛿**: -0.17 (-8%) | NA | NA | 60s. 1W. 808nm. SLA surface | Profilometer |
|  | Diode-Epic10 | Lee. 2023 | **T**: 1.247  **C**:1.316  **𝛿**: -0.069 (-5%) | **T**: 1.196  **C**: 1.485  **𝛿**: -0.289(-19%) | NA | 10s. 940nm. 2W. 100mJ. 10Hz. SLA surface | Microscope |
|  | Diode-Epic10 | Lee. 2023 | **T**: 1.342  **C**:1.534  **𝛿**: -0.19 (-13%) | **T**: 1.433  **C**: 1.551  **𝛿**: -0.12 (-8%) | NA | 10s. 940nm. 2W. 100mJ. 10Hz. Femtosecond laser surface treatment | Microscope |
|  | Diode-Epic10 | Kim. 2020 | **T**: 0.174±0.043  **C**: 0.177±0.018  **𝛿**: 0.003 (-2%) | NA | NA | 2W. 940nm. SLA surface | Microscope |
|  | Diode-Epic10 | Kim. 2020 | **T**: 0.182±0.070  **C**: 0.177±0.018  **𝛿**: 0.005 (3%) | NA | NA | 1W. 940nm. SLA surface | Microscope |
|  | Diode-Epic10 | Kim. 2020 | **T**: 0.267±0.095  **C**: 0.177±0.018  **𝛿**: 0.09 (51%) | NA | NA | 3W. 940nm. SLA surface | Microscope |
|  | Er. Cr:YSGG | Ercan. 2013 | **T**: 0.968  **C**:1.292  **𝛿**: -0.324 (-25%) | NA | NA | 45s. 3W. 25Hz. distance 2mm. RBT surface | Profilometer |
|  | Er. Cr:YSGG | Ercan. 2013 | **T**:1.188 **C**:1.292  **𝛿**: -0.104 (-8%) | NA | NA | 15s. 2W. 25Hz. distance 6mm. RBT surface | Profilometer |
|  | Er.Cr:YSGG | Ercan. 2013 | **T**:1.188 **C**:1.292  **𝛿**:-0.104 (-8%) | NA | NA | 15s. 1W. 20Hz. distance 2mm. RBT surface | Profilometer |
|  | Er.Cr:YSGG | Ercan. 2013 | **T**:1.215  **C**:1.292  **𝛿**:-0.077 (-6%) | NA | NA | 45s. 1W. 30Hz. distance 6mm. RBT surface | Profilometer |
|  | Er.Cr:YSGG | Ercan. 2013 | **T**:1.267  **C**:1.292  **𝛿**: -0.03 (-2%) | NA | NA | 30s. 2W. 30Hz. distance 2mm. RBT surface | Profilometer |
|  | Er.Cr:YSGG | Ercan. 2013 | **T**:1.289  **C**:1.292  **𝛿**:-0.003 (0%) | NA | NA | 15s. 3W. 30Hz. distance 4mm. RBT surface | Profilometer |
|  | Er.Cr:YSGG | Ercan. 2013 | **T**:1.32  **C**:1.292  **𝛿**:0.03 (2%) | NA | NA | 30s. 3W. 20Hz. distance 6mm. RBT surface | Profilometer |
|  | Er.Cr:YSGG | Ercan. 2013 | **T**:1.427  **C**:1.292  **𝛿**:0.13 (10%) | NA | NA | 45s. 2W. 20Hz. distance 4mm. RBT surface | Profilometer |
|  | Er.Cr:YSGG | Ercan. 2013 | **T**:1.443  **C**:1.292  **𝛿**: 0.15 (12%) | NA | NA | 30s. 1W. 25Hz. distance 4mm. RBT surface | Profilometer |
|  | Er.Cr:YSGG | Khalil. 2023 | **T**: 3.56±0.2**  **C**: 2.81±0.10  **𝛿**: 0.75% (27%) | NA | NA | 30Hz. 2780nm. 120mJ. SLA surface | Profilometer |
|  | Er.Cr:YSGG | Lee. 2023 | **T**:1.38*  **C**:1.316  **𝛿**:0.07 (5%) | **T**: 1.552  **C**: 1.485  **𝛿**: 0.07 (5%) | NA | 10s. 2780nm. 2W. 300mJ. 10Hz. SLA surface | Microscope |
|  | Er.Cr:YSGG | Lee. 2023 | **T**:1.473*  **C**:1.534  **𝛿**:-0.06 (-4%) | **T**: 1.735  **C**: 1.551  **𝛿**: 0.18 (12%) | NA | 10s. 2780nm. 2W. 300mJ. 10Hz. Femtosecond laser surface treatment | Microscope |
|  | Er:YAG | Duarte. 2009 | **T**: 0.68±0.06  **C**: 0.70±0.7  **𝛿**: -0.02 (-3%) | NA | NA | 45°. 1-15Hz. 2940nm. 60-500mJ. 120mJ/pulse. SLA surface | Profilometer |
|  | K2Mobile | Kim. 2020 | **T**: 0.147±0.007  **C**: 0.177±0.018  **𝛿**: -0.03 (-17%) | NA | NA | 1W. 980nm. SLA surface | Microscope |
|  | K2Mobile | Kim. 2020 | **T**: 0.183±0.073  **C**: 0.177±0.018  **𝛿**: 0.006 (3%) | NA | NA | 3W. 980nm. SLA surface | Microscope |
|  | K2Mobile | Kim. 2020 | **T**: 0.204±0.029  **C**: 0.177±0.018  **𝛿**: 0.027 (15%) | NA | NA | 2W. 980nm. SLA surface | Microscope |
|  | Nd:YAG | Li. 2022 | NA | **T**: 1.60±0.09 **C**: 1.58±0.05  **𝛿**: 0.02 (1%) | NA | 45s. 1W. 15Hz. distance 2mm. 1064nm. SLA surface | Microscope |
|  | Nd:YAG | Li. 2022 | NA | **T**: 1.63±0.16 **C**: 1.58±0.05  **𝛿**: 0.05 (3%) | NA | 45s. 0.5W. 15Hz. distance 2mm. 1064nm. SLA surface | Microscope |
|  | Saeshin | Kim. 2020 | **T**: 0.144±0.067 **C**: 0.177±0.018  **𝛿**: -0.033 (-19%) | NA | NA | 1W. 980nm. SLA surface | Microscope |
|  | Saeshin | Kim. 2020 | **T**: 0.158±0.056 **C**: 0.177±0.018  **𝛿**: -0.019 (-11%) | NA | NA | 2W. 980nm. SLA surface | Microscope |
|  | Saeshin | Kim. 2020 | **T**: 0.166±0.040 **C**: 0.177±0.018  **𝛿**: -0.011 (-6%) | NA | NA | 3W. 980nm. SLA surface | Microscope |
| Toothbrush |  | Park. 2012 | **T**: 1.54±0.27  **C**: 2.25±0.15  **𝛿**: -0.71 (-32%) | **T**:1.60±0.21 **C**:2.30±0.09  **𝛿**: -0.7 (-30%) | **T**: 6.26±0.99 **C**: 9.11±0.54  **𝛿**: -2.85 (-31%) | SLA surface | Microscope |
| Toothbrush |  | Park. 2013 | **T**: 2.58±0.53  **C**: 2.75±0.55  **𝛿**: -0.17 (-6%) | **T**: 2.94±0.33  **C**: 2.75±0.20  **𝛿**: 0.19 (7%) | **T**: 7.25±1.39  **C**: 8.02±1.80  **𝛿**: -0.77 (-10%) | RBM surface | Microscope |
| Ultrasonic | Carbon | Park. 2013 | **T**: 3.06±0.77 **C**: 2.75±0.55  **𝛿**: 0.31 (11%) | **T**: 3.07±0.31 **C**: 2.75±0.20  **𝛿**: 0.32 (12%) | **T**: 7.78±0.87 **C**: 8.02±1.80  **𝛿**: -0.24 (-3%) | RBM surface | Microscope |
|  | Carbon | Sahrmann. 2021 | **T**: 1.03±0.13*  **C**: 1.37±0.14  **𝛿**: -0.34 (-25%) | NA | **T**: 5.15±0.75* **C**: 7.33±0.56  **𝛿**: -2.18 (-30%) | 30s. 180Hz. pressure100g. Iniciell surface | Profilometer |
|  | Carbon | Sahrmann. 2021 | **T**: 1.10±0.17*  **C**: 1.30±0.14  **𝛿**: -0.2 (-15%) | NA | **T**: 5.48±0.83* **C**: 7.33±0.82  **𝛿**: -1.85 (-25%) | 30s. 180Hz. pressure100g. SLA surface | Profilometer |
|  | Metal | Sahrmann. 2021 | **T**: 0.57±0.09*  **C**: 1.37±0.14  **𝛿**: -0.8 (-58%) | NA | **T**: 3.40±0.61 * **C**: 7.33±0.56  **𝛿**: -3.93 (-54%) | 30s. 180Hz. pressure100g. Iniciell surface | Profilometer |
|  | Metal | Sahrmann. 2021 | **T**: 0.58±0.14*  **C**: 1.30±0.14  **𝛿**: -0.72 (-55%) | NA | **T**: 3.34±0.76* **C**: 7.33±0.82  **𝛿**: -3.99 (-54%) | 30s. 180Hz. pressure100g. SLA surface | Profilometer |
|  | Metal (EMS Tip) | Park. 2012 | **T**: 1.63±0.55  **C**: 2.25±0.15  **𝛿**: -0.62 (-28%) | **T**: 1.72±0.36 **C**: 2.30±0.09  **𝛿**: -0.58 (-25%) | **T**: 5.47±1.93 **C**: 9.11±0.54  **𝛿**: -3.64 (-40%) | 40sec. 20strokes. 90°. power 3. SLA surface | Microscope |
|  | Metal (EMS Tip) | Park. 2013 | **T**: 2.44±0.70*  **C**: 2.75±0.55  **𝛿**: -0.31 (-11%) | **T**: 2.41±0.26* **C**: 2.75±0.20 **𝛿**: -0.34 (-12%) | **T**: 6.13±1.87* **C**: 8.02±1.80  **𝛿**: -1.89 (-24%) | Power 3. 25-32Hz. RBM surface | Microscope |
|  | Metal (Satelec Tip) | Park. 2012 | **T**: 2.01±0.27  **C**: 2.25±0.15  **𝛿**: -0.24 (-11%) | **T**: 2.09±0.10 **C**: 2.30±0.09  **𝛿**: -0.21 (-9%) | **T**: 7.29±1.21 **C**: 9.11±0.54  **𝛿**: -1.82 (-20%) | 40s. 20strokes. 90°. power 3. SLA surface | Microscope |
|  | Metal (Satelec Tip) | Park. 2013 | **T**: 2.55±0.77 **C**:2.75±0.55  **𝛿**: -0.2 (-7%) | **T**: 2.70±0.18 **C**: 2.75±0.20  **𝛿**: -0.05 (-2%) | **T**: 6.84±1.46 **C**: 8.02±1.80  **𝛿**: -1.18 (-15%) | Power 3. 27-33Hz. RBM surface | Microscope |
|  | Plastic | Park. 2013 | **T**: 2.67±0.62  **C**: 2.75±0.55  **𝛿**: -0.08 (-3%) | **T**: 2.85±0.20 **C**: 2.75±0.20  **𝛿**: 0.1 (4%) | **T**: 7.08±1.62 **C**: 8.02±1.80  **𝛿**: -0.94 (-10%) | RBM surface | Microscope |
|  | Plastic | Sahrmann. 2021 | **T**: 0.88±0.17*  **C**: 1.37±0.14  **𝛿**: -0.49 (-36%) | NA | **T**: 4.48±0.69* **C**: 7.33±0.56  **𝛿**: -2.85 (-39%) | 30s. 180Hz. pressure100g. Iniciell surface | Profilometer |
|  | Plastic | Sahrmann. 2021 | **T**: 0.99±0.25*  **C**: 1.30±0.14  **𝛿**: -0.31 (-24%) | NA | **T**: 4.03±0.83* **C**: 7.33±0.82  **𝛿**: -3.3 (-45%) | 30s. 180Hz. pressure100g. SLA surface | Profilometer |
|  | Plastic (EMS Tip) | Park. 2012 | **T**: 1.95±0.30  **C**: 2.25±0.15  **𝛿**: -0.345 (-15%) | **T**: 2.10±0.18 **C**: 2.30±0.09  **𝛿**: -0.2 (-9%) | **T**: 7.18±0.79 **C**: 9.11±0.54  **𝛿**: -1.93 (-21%) | 40s. 20strokes. 90°. power3. SLA surface | Microscope |
|  | Plastic (Satelec Tip) | Park. 2012 | **T**: 2.09±0.30  **C**: 2.25±0.15  **𝛿**: -0.16 (-7%) | **T**: 2.11±0.19 **C**: 2.30±0.09  **𝛿**: -0.19 (-8%) | **T**: 7.22±1.00* **C**: 9.11±0.54  **𝛿**: -1.89 (-21%) | 40s. 20strokes. 90°. power3. SLA surface | Microscope |
|  | Resin | Sahrmann. 2021 | **T**: 1.37±0.17  **C**: 1.37±0.14  **𝛿**: 0 (0%) | NA | **T**: 6.90±0.78 **C**: 7.33±0.56  **𝛿**: -0.43 (-6%) | 30s. 180Hz. pressure100g. Iniciell surface | Profilometer |
|  | Resin | Sahrmann. 2021 | **T**: 1.33±0.21  **C**: 1.30±0.14  **𝛿**: 0.03 (2%) | NA | **T**: 6.94±1.22 **C**: 7.33±0.82  **𝛿**: -0.39 (-5%) | 30s. 180Hz. pressure100g. SLA surface | Profilometer |
|  | Titanium | Faccioni. 2021 | **T**:0.375±0.1258* **C**:0.8432±0.0073  **𝛿**: -0.468 (-56%) | NA | NA | 40time. power3. 25-32Hz. pressure30g. LaserPass surface | Profilometer |
|  | Titanium | Faccioni. 2021 | **T**:0.3031±0.113* **C**:0.5295±0.0491  **𝛿**: -0.226 (-43%) | NA | NA | 40time. power3. 25-32Hz. pressure30g. Sand-Blasted surface | Profilometer |
|  | Titanium | Sahrmann. 2021 | **T**: 0.76±0.18*  **C**: 1.37±0.14  **𝛿**: -0.61 (-45%) | NA | **T**: 4.43±0.87* **C**: 7.33±0.56  **𝛿**: -2.9 (-40%) | 30s. 180Hz. pressure100g. Iniciell surface | Profilometer |
|  | Titanium | Sahrmann. 2021 | **T**: 0.74±0.23*  **C**: 1.30±0.14  **𝛿**: -0.56 (-43%) | NA | **T**: 4.89±0.96* **C**: 7.33±082  **𝛿**: -2.44 (-33%) | 30s. 180Hz. pressure100g. SLA surface | Profilometer |

T= test group. C= control group. 𝛿= difference between test and control group. NA: Not Available *= p< 0.05. **=p>0.01. SLA= sandblasted acid-etched. RBM= treated by resorbable blast media

Supplementary Table 5. Surface roughness values on machined titanium surfaces (µm. mean ± SD)

| **Instrument** | **Type** | **Article** | **Ra** | **Sa** | **Rz** | **Protocol** | **Measurement** |
| --- | --- | --- | --- | --- | --- | --- | --- |
| Air-Polishing | Erythritol14μm | Hui. 2021 | NA | **T**: 1.1525  **C**: 1.19  **𝛿**: -0.0375 (-3%) | NA | 20s.distance 10mm. | Profilometer |
|  | Glycine | Biazussi. 2019 | **T**: 0.1189±0.049 **C**: 0.1129±0.033 **𝛿**: 0.006 (5%) | NA | NA | 20s. distance 4mm. | Profilometer |
|  | Glycine | Cafiero. 2016 | **T**: 0.16±0.06  **C**: 0.23±0.14  **𝛿**: -0.07 (-30%) | NA | **T**: 1.05±0.83** **C**: 3.05±1.70  **𝛿**: -2 (-66%) | 10s. high power. distance 5mm | Profilometer |
|  | Glycine | Cafiero. 2016 | **T**: 0.23±0.18 **C**: 0.23±0.14  **𝛿**: 0 (0%) | NA | **T**: 1.80±2.33 **C**: 3.05±1.70  **𝛿**: -1.25 (-41%) | 10s. low power. distance 5mm | Profilometer |
|  | Glycine | Huang. 2019 | **T**: 0.09±0.01 **C**: 0.10±0.02  **𝛿**: -0.01 (-10%) | NA | NA | Medium power. distance 0.5-1cm | Profilometer |
|  | SodiumBicarbonate | Biazussi. 2019 | **T**:0.2126±0.058* **C**:0.1129±0.033  **𝛿**: 0.1 (89%) | NA | NA | 20s. distance 4mm | Profilometer |
|  | SodiumBicarbonate | Duarte. 2009 | **T**: 0.20±0.006 **C**: 0.18±0.002  **𝛿**: 0.02 (11%) | NA | NA | Medium power. distance 5mm | Profilometer |
|  | SodiumBicarbonate | Kister. 2017 | **T**: 0.18±0.06 **C**: 0.19±0.04  **𝛿**: -0.01 (-5%) | NA | NA | 20s. pressure 0.85±0.30N. distance 5mm | Profilometer |
|  | SodiumBicarbonate | Kister. 2017 | **T**: 0.19±0.11 **C**: 0.18±0.03  **𝛿**: 0.01 (6%) | NA | NA | 20s. pressure 0.69±0.23N. distance 5mm | Profilometer |
| Brush | Metal | Kim. 2019 | **T**: 0.47±0.13** **C**: 0.26±0.09  **𝛿**: 0.21 (81%) | **T**: 0.56±0.20** **C**: 0.28±0.09  **𝛿**: 0.28 (100%) | **T**: 2.01±0.47** **C**: 1.14±0.39  **𝛿**: 0.87 (76%) | 40s | Microscope |
|  | Nylon | Cafiero. 2016 | **T**: 0.25±0.12 **C**: 0.23±0.14  **𝛿**: 0.02 (9%) | NA | **T**: 2.15±1.23 **C**: 3.05±1.70  **𝛿**: -0.9 (-30%) | 5s. 800rpm. Perlite | Profilometer |
|  | Nylon | Cafiero. 2016 | **T**: 0.30±0.15 **C**: 0.23±0.14  **𝛿**: 0.07 (30%) | NA | **T**: 3.70±1.13 **C**: 3.05±1.70  **𝛿**: 0.65 (21%) | 5s. 800rpm. Detartrine | Profilometer |
|  | Nylon | Kim. 2019 | **T**: 0.61±0.19** **C**: 0.26±0.09  **𝛿**: 0.35 (135%) | **T**: 0.63±0.20** **C**: 0.28±0.09  **𝛿**: 0.35 (125%) | **T**: 2.09±0.81** **C**: 1.14±0.39  **𝛿**: 0.95 (83%) | 40s | Microscope |
|  | Nylon | Sawase. 2005 | **T**: 0.019  **C**:0.017  **𝛿**:0.002 (12%) | NA | NA | 2min. pressure 500g. TiN surface | Profilometer |
|  | Nylon | Sawase. 2005 | **T**:0.097** **C**:0.017  **𝛿**:0.08 (471%) | NA | NA | 2min. pressure 500g. Ti surface | Profilometer |
|  | Titanium | Lang. 2016 | **T**: 1.37±0.124**  **C**:1.09±0.098  **𝛿**: 0.28 (26%) | NA | NA | 60s | Profilometer |
|  | Titanium | Park. 2015 | **T**: 0.39±0.15 **C**: 0.72±0.50  **𝛿**: -0.33 (-46%) | **T**: 0.41±0.14 **C**: 0.82±0.54  **𝛿**: -0.41 (-50%) | **T**: 1.52±0.56 **C**: 2.59±1.48  **𝛿**: -1.07 (-41%) | 40s. 300rpm | Microscope |
|  | Titanium | Park. 2015 | **T**: 0.37±0.08 **C**: 0.37±0.08  **𝛿**: 0 (0%) | NA | NA | 40s. 300rpm | Profilometer |
| Chlorexidine | 1ml of 0.12% | Batsukh. 2017 | **T**: 0.2028±0.01 **C**: 0.2193±0.04  **𝛿**: -0.01 (-5%) | NA | NA | 24h | Profilometer |
| Cold Atmospheric Plasma |  | Hui. 2021 | NA | **T**: 1.225  **C**: 1.19  **𝛿**: 0.035 (3%) | NA | Distance 5mm. 1.4Hz. 10kV. 2°C | Profilometer |
| Cup | Rubber | Batsukh. 2017 | **T**: 0.1772±0.04 **C**: 0.2193±0.04 **𝛿**: -0.04 (-18%) | NA | NA | 60s. paste | Profilometer |
|  | Rubber | Cafiero. 2016 | **T**: 0.28±0.16  **C**: 0.23±0.14  **𝛿**: 0.05 (22%) | NA | **T**: 2.05±1.50 **C**: 3.05±1.70  **𝛿**: -1 (-33%) | 5s. 800rpm. Perlite | Profilometer |
|  | Rubber | Cafiero. 2016 | **T**: 0.33±0.19 **C**: 0.23±0.14  **𝛿**: 0.1 (43%) | NA | **T**: 2.45±1.43 **C**: 3.05±1.70  **𝛿**: -0.6 (-20%) | 5s. 800rpm. Detartrine | Profilometer |
|  | Rubber | Kister. 2017 | **T**: 0.14±0.05 **C**: 0.18±0.03  **𝛿**: -0.04 (-22%) | NA | NA | 20s. 500rpm. pressure 0.05±0.03N. fluoride paste | Profilometer |
|  | Rubber | Kister. 2017 | **T**: 0.21±0.04 **C**: 0.19±0.04  **𝛿**: 0.02 (11%) | NA | NA | 20s. 500rpm. pressure 0.08±0.03N. fluoride paste | Profilometer |
| Curette | Metal | Bertoldi. 2016 | **T**: 0.185**  **C**: 0.15  **𝛿**:0.035 (23%) | NA | NA | Pressure 100g | Profilometer |
|  | Metal | Duarte. 2009 | **T**: 0.38±0.08* **C**: 0.20±0.02  **𝛿**: 0.18 (90%) | NA | NA | 30strokes. 70° | Profilometer |
|  | Metal | Kister. 2017 | **T**: 0.41±0.13 **C**: 0.19±0.04  **𝛿**: 0.22 (116%) | NA | NA | 20times. pressure 1.95±0.54N | Profilometer |
|  | Metal | Kister. 2017 | **T**: 0.66±0.09 **C**: 0.18±0.03  **𝛿**: 0.48 (267%) | NA | NA | 20times. pressure 2.25±0.62N | Profilometer |
|  | Plastic | Duarte. 2009 | **T**: 0.24±0.02 **C**: 0.19±0.02  **𝛿**: 0.05 (26%) | NA | NA | 30strokes. 70° | Profilometer |
|  | Plastic | Kister. 2017 | **T**: 0.20±0.20 **C**: 0.19±0.04  **𝛿**: 0.01 (5%) | NA | NA | 20times. pressure 2.54±1.23N | Profilometer |
|  | Plastic | Kister. 2017 | **T**: 0.23±0.05 **C**: 0.18±0.03  **𝛿**: 0.05 (28%) | NA | NA | 20times. pressure 2.74±0.69N | Profilometer |
|  | Plastic | Lang. 2016 | **T**: 0.61±0.042 **C**: 1.09±0.098  **𝛿**: -0.48 (-44%) | NA | NA | 20strokes | Profilometer |
|  | Plastic | Lang. 2016 | **T**: 0.68±0.062 **C**: 1.09±0.098  **𝛿**: -0.41 (-38%) | NA | NA | 100strokes | Profilometer |
|  | Plastic | Unursaikhan. 2012 | **T**: 0.20 ± 0.04 **C**:0.24±0.07  **𝛿**: -0.04 (-17%) | NA | **T**: 1.38±0.51 **C**: 1.49±0.50  **𝛿**: -0.11 (-7%) | 30strokes. 45° | Profilometer |
|  | Reinforced carbon plastic | Huang. 2019 | **T**: 0.14±0.02 **C**: 0.10±0.02  **𝛿**: 0.04 (40%) | NA | NA | Overlapping strokes | Profilometer |
|  | Titanium | Bertoldi. 2016 | **T**:0.106**  **C**: 0.142  **𝛿**:-0.036 (-25%) | NA | NA | Pressure 100g | Profilometer |
|  | Titanium | Gehrke. 2018 | NA | **T**: 0.65 ± 0.04 **C**: 0.70 ± 0.05  **𝛿**: -0.05 (-7%) | NA | 20strokes | Profilometer |
|  | Titanium | Huang. 2019 | **T**: 0.45±0.11* **C**: 0.10±0.02  **𝛿**: 0.35 (350%) | NA | NA | Overlapping strokes | Profilometer |
|  | Titanium | Lang. 2016 | **T**: 1.38±0.219 **C**: 1.09±0.098  **𝛿**: 0.29 (27%) | NA | NA | 20strokes | Profilometer |
|  | Titanium | Lang. 2016 | **T**: 1.71±0.048 **C**: 1.09±0.098  **𝛿**: 0.62 (57%) | NA | NA | 100strokes | Profilometer |
| Laser | Diode | Lang. 2016 | **T**:1.26±0.113 **C**: 1.09±0.098  **𝛿**: 0.17 (16%) | NA | NA | 60s. 1.4W | Profilometer |
|  | Diode-Epic10 | Lee. 2023 | **T**: 0.657  **C**:0.58  **𝛿**:0.077 (13%) | **T**: 0.251  **C**: 0.192  **𝛿**: 0.059 (31%) | NA | 10s. 940nm. 2W. 100mJ. 10Hz | Microscope |
|  | Er.Cr:YSGG | Lee. 2023 | **T**:1.034  **C**:0.58  **𝛿**:0.454 (78%) | **T**: 1.771  **C**: 0.192  **𝛿**: 1.579 (822%) | NA | 10s. 2780nm. 2W. 300mJ. 10Hz | Microscope |
|  | Er:YAG | Duarte. 2009 | **T**:0.23±0.06 **C**:0.18±0.02  **𝛿**: 0.05 (28%) | NA | NA | 45°. 1.15Hz. 2940nm. 60-500mJ. 120mJ/pulse | Profilometer |
|  | GaAlAs | Batsukh. 2017 | **T**: 0.2119±0.02 **C**: 0.2193±0.04  **𝛿**: -0.0074 (-3%) | NA | NA | 60s. 940nm. 100mJ/pulse | Profilometer |
| Scaler | Metal | Fakhravar. 2023 | **T**: 3.2296±0.68 **C**: 1.1828±0.28  **𝛿**: 2.047 (173%) | NA | NA | 5stroke | Profilometer |
|  | Metal | Sawase. 2005 | **T**:0.036  **C**: 0.017  **𝛿**: 0.019 (112%) | NA | NA | 20strokes. pressure 500g. TiN surface | Profilometer |
|  | Metal | Sawase. 2005 | **T**:0.0356* **C**:0.017  **𝛿**: 0.018 (106%) | NA | NA | 20strokes. pressure 500g. Ti surface | Profilometer |
|  | Plastic | Fakhravar. 2023 | **T**: 2.1522±0.77 **C**: 1.1828±0.28 **𝛿**: 0.97 (82%) | NA | NA | 5stroke | Profilometer |
|  | Plastic | Gehrke. 2018 | NA | **T**: 0.64 ± 0.04 **C**: 0.70 ± 0.05  **𝛿**: -0.06 (-9%) | NA | 20strokes | Profilometer |
|  | Plastic | Sawase. 2005 | **T**:0.017  **C**:0.017  **𝛿**: 0 (0%) | NA | NA | 20strokes. pressure 500g. TiN surface | Profilometer |
|  | Plastic | Sawase. 2005 | **T**:0.024  **C**:0.017  **𝛿**:0.007 (41%) | NA | NA | 20strokes. pressure 500g. Ti surface | Profilometer |
| Toothbrush |  | Park. 2012 | **T**: 0.40±0.22  **C**: 0.67±0.52  **𝛿**: -0.27 (-40%) | **T**: 0.48±0.17 **C**: 0.77±0.55  **𝛿**: -0.29 (-38%) | **T**: 2.23±1.02 **C**: 2.38±1.31  **𝛿**: -0.15 (-6%) | 60s | Microscope |
| Toothbrush |  | Sawase. 2005 | **T**: 0.019 **C**:0.017  **𝛿**:0.002 (12%) | NA | NA | pressure 350g. TiN surface | Profilometer |
| Toothbrush |  | Sawase. 2005 | **T**:0.069 **C**:0.017  **𝛿**:0.052 (306%) | NA | NA | pressure 350g. Ti surface | Profilometer |
| Ultrasonic | Bronze | Chun. 2017 | **T**:0.5  **C**:0.4  **𝛿**:0.1 (25%) | NA | NA | 30s. 30°. 30%power. pressure 40g | Microscope |
|  | Carbon | Huang. 2019 | **T**: 0.18±0.03 **C**: 0.10±0.02  **𝛿**: 0.08 (80%) | NA | NA | Power2 | Profilometer |
|  | Carbon | Sahrmann. 2021 | **T**: 0.35±0.05 **C**: 0.40±0.07  **𝛿**: -0.05 (-13%) | NA | **T**: 2.04±0.30* **C**: 2.48±0.40  **𝛿**: -0.44 (-18%) | 30s. 180Hz. pressure100g No treated surface | Profilometer |
|  | Carbon | Sahrmann. 2021 | **T**:0.06±0.02 **C**: 0.04±0.01  **𝛿**: 0.02 (50%) | NA | **T**: 0.40±0.16* **C**: 0.02±0.07  **𝛿**: 0.38 (1900%) | 30s. 180Hz. pressure100g. Machined surface | Profilometer |
|  | Copper | Chun. 2017 | **T**:0.5  **C**:0.04  **𝛿**: 0.01 (25%) | NA | NA | 30s. 30°. 40%power. pressure 40g | Microscope |
|  | Metal | Batsukh. 2017 | **T**: 0.5174±0.12** **C**: 0.2193±0.04  **𝛿**: 0.298 (136%) | NA | NA | 60s | Profilometer |
|  | Metal | Gehrke. 2018 | NA | **T**: 4.00 ± 1.68* **C**: 0.70 ± 0.05  **𝛿**: 3.3 (471%) | NA | 30Hz | Profilometer |
|  | Metal | Kister. 2017 | **T**: 0.25±0.10 **C**: 0.18±0.03  **𝛿**: 0.07 (39%) | NA | NA | 20times. pressure 1.90±0.79N | Profilometer |
|  | Metal | Kister. 2017 | **T**: 0.28±0.08 **C**: 0.19±0.04  **𝛿**: 0.09 (47%) | NA | NA | 20s. power2. pressure 2.15±0.69N | Profilometer |
|  | Metal | Sahrmann. 2021 | **T**: 0.54±0.10* **C**: 0.40±0.07  **𝛿**: 0.14 (35%) | NA | **T**: 2.82±0.42* **C**: 2.48±0.40  **𝛿**: 0.34 (14%) | 30s. 180Hz. pressure100g. No treated surface | Profilometer |
|  | Metal | Sahrmann. 2021 | **T**: 0.42±0.08* **C**: 0.04±0.01  **𝛿**: 0.38 (950%) | NA | **T**: 2.30±0.49 * **C**: 0.02±0.07  **𝛿**: 2.28 (11400%) | 30s. 180Hz. pressure100g. Machined surface | Profilometer |
|  | Metal | Sawase. 2005 | **T**:0.111** **C**:0.017  **𝛿**:0.094 (553%) | NA | NA | 2min. TiN surface | Profilometer |
|  | Metal | Sawase. 2005 | **T**:1.337** **C**:0.017  **𝛿**:1.32 (7765%) | NA | NA | 2min. Ti surface | Profilometer |
|  | Metal (316L. Catatec) | Chun. 2017 | **T**:2.1*  **C**:0.4  **𝛿**:1.7 (425%) | NA | NA | 30s. 30°. 40%power. pressure 40g | Microscope |
|  | Metal (EMS Tip) | Park. 2012 | **T**: 0.86±0.21 **C**: 0.67±0.52  **𝛿**: 0.19 (28%) | **T**: 0.95±0.18 **C**: 0.77±0.55  **𝛿**: 0.18 (23%) | **T**: 3.75±0.62 **C**: 2.38±1.31  **𝛿**: 1.37 (58%) | 20strokes. 40s. 90°. power3 | Microscope |
|  | Metal (Satelec Tip) | Park. 2012 | **T**: 0.76±0.30 **C**: 0.67±0.52  **𝛿**: 0.09 (13%) | **T**: 0.78±0.17 **C**: 0.77±0.55  **𝛿**: 0.01 (1%) | **T**: 3.06±0.89 **C**: 2.38±1.31  **𝛿**: 0.68 (29%) | 20strokes. 40s. 90°. power3 | Microscope |
|  | Metal (Satelec) | Chun. 2017 | **T**:5.7*  **C**:0.4  **𝛿**:5.3 (1325%) | NA | NA | 30s. 30°. 40%power. pressure 40g | Microscope |
|  | Metal (US Satelec - Tip EMS) | Unursaikhan.2012 | **T**: 0.36±0.04 **C**: 0.24±0.07  **𝛿**: 0.12 (50%) | NA | **T**: 2.54±0.50 **C**: 1.49±0.50  **𝛿**: 1.05 (70%) | 45°. high power. 27-32Hz | Profilometer |
|  | Metal (US Satelec - Tip EMS) | Unursaikhan.2012 | **T**: 0.39 ±0.02 **C**: 0.24±0.07  **𝛿**: 0.15 (63%) | NA | **T**: 2.82±0.78 **C**: 1.49±0.50  **𝛿**: 1.33 (89%) | 45°. high power. 27-32Hz | Profilometer |
|  | Metal (US Yoshida - Tip B&L Biotech) | Unursaikhan.2012 | **T**: 0.22±0.05. **C**: 0.24±0.07  **𝛿**: -0.02 (-8%) | NA | **T**: 1.59±0.54 **C**: 1.49±0.50  **𝛿**: 0.1 (7%) | 30s. 15°. high power. 25Hz | Profilometer |
|  | Metal (US Yoshida - Tip B&L Biotech) | Unursaikhan.2012 | **T**: 0.23±0.06. **C**: 0.24±0.07  **𝛿**: -0.01 (-4%) | NA | **T**: 1.79±1.51 **C**: 1.49±0.50  **𝛿**: 0.3 (20%) | 30s. 45°. high power. 25Hz | Profilometer |
|  | Metal (US Yoshida - Tip EMS) | Unursaikhan. 2012 | **T**: 0.34 ±0.06 **C**: 0.24±0.07  **𝛿**: 0.1 (42%) | NA | **T**: 2.4±0.53 **C**: 1.49±0.50  **𝛿**: 0.91 (61%) | 30s. 45°. high power. 25Hz | Profilometer |
|  | Metal (US Yoshida - Tip EMS) | Unursaikhan. 2012 | **T**: 0.34±0.03 **C**: 0.24±0.07  **𝛿**: 0.1 (42%) | NA | **T**: 2.54±0.79 **C**: 1.49±0.50  **𝛿**: 1.05 (70%) | 30s. 15°. high power. 25Hz | Profilometer |
|  | Plastic | Bertoldi. 2016 | **T**:0.147  **C**:0.15  **𝛿**:-0.003 (-2%) | NA | NA | Pressure 100g | Profilometer |
|  | Plastic | Kister. 2017 | **T**: 0.18±0.04 **C**: 0.19±0.04  **𝛿**: -0.01 (-5%) | NA | NA | 20s. power2. pressure 0.08±0.05N | Profilometer |
|  | Plastic | Kister. 2017 | **T**: 0.19±0.05  **C**: 0.18±0.03  **𝛿**: 0.01 (6%) | NA | NA | 20s. power2. pressure 0.05±0.02N | Profilometer |
|  | Plastic | Sahrmann. 2021 | **T**: 0.39±0.07 **C**: 0.40±0.07  **𝛿**: -0.01 (-3%) | NA | **T**: 2.26±0.45* **C**: 2.48±0.40  **𝛿**: -0.22 (-9%) | 30s. 180Hz. pressure100g. No treated surface | Profilometer |
|  | Plastic | Sahrmann. 2021 | **T**: 0.05±0.03 **C**: 0.04±0.01  **𝛿**: 0.01 (25%) | NA | **T**: 0.36±0.16 **C**: 0.02±0.07  **𝛿**: 0.34 (1700%) | 30s. 180Hz. pressure100g. Machined surface | Profilometer |
|  | Plastic (EMS Tip) | Park. 2012 | **T**: 0.64±0.28 **C**: 0.67±0.52  **𝛿**: -0.03 -(4%) | **T**: 0.71±0.23 **C**: 0.77±0.55  **𝛿**: -0.06 (-8%) | **T**: 3.18±0.51 **C**: 2.38±1.31  **𝛿**: 0.8 (34%) | 20strokes. 40s. 90°. power3 | Microscope |
|  | Plastic (Satelec Tip) | Park. 2012 | **T**: 0.61±0.33 **C**: 0.67±0.52  **𝛿**: -0.06 (-9%) | **T**: 0.62±0.25 **C**:0.77±0.55  **𝛿**: -0.15 (-19%) | **T**: 2.86±0.86 **C**: 2.38±1.31  **𝛿**: 0.48 (20%) | 2ostrokes. 40s. 90°. power3 | Microscope |
|  | Resin | Sahrmann. 2021 | **T**: 0.37±0.04 **C**: 0.40±0.07  **𝛿**: -0.03 (8%) | NA | **T**: 2.22±0.31* **C**: 2.48±0.40  **𝛿**: -0.26 (-10%) | 30s. 180Hz. pressure100g. No treated surface | Profilometer |
|  | Resin | Sahrmann. 2021 | **T**: 0.04±0.02 **C**: 0.04±0.01  **𝛿**: 0 (0%) | NA | **T**: 0.30±0.11 **C**: 0.02±0.07  **𝛿**: 0.28 (1400%) | 30s. 180Hz. pressure100g. Machined surface | Profilometer |
|  | Titanium | Faccioni. 2021 | **T**:0.167±0.0465*  **C**: 0.131±0.0374  **𝛿**: 0.036 (28%) | NA | NA | 40times. power3. 25-32Hz. pressure 30g. Machined surface | Profilometer |
|  | Titanium | Faccioni. 2021 | **T**:0.1457±0.2883* **C**:0.2056±0.0223  **𝛿**: -0.06 (-29%) | NA | NA | 40time. power3. 25-32Hz. pressure30g: LaserNonpass surface | Profilometer |
|  | Titanium | Sahrmann. 2021 | **T**: 0.59±0.18* **C**: 0.40±0.07  **𝛿**: 0.19 (48%) | NA | **T**: 3.18±0.83** **C**: 2.48±0.40  **𝛿**: 0.7 (28%) | 30s. 180Hz. pressure100g. No treated surface | Profilometer |
|  | Titanium | Sahrmann. 2021 | **T**: 0.36±0.18* **C**: 0.04±0.01  **𝛿**: 0.32 (800%) | NA | **T**: 2.21±0.90** **C**: 0.02±0.07  **𝛿**: 2.19 (10950%) | 30s. 180Hz. pressure100g. | Profilometer |

T= test group. C= control group. 𝛿= difference between test and control group. NA: Not Available *= p< 0.05. **=p>0.0. TiN= Titanium Nitride-coated. Ti= pure titanium

Supplementary Table 6. Surface roughness values on zirconia surfaces (µm. mean ± SD)

| **Instrument** | **Type** | **Article** | **Ra** | **Sa** | **Protocol** | **Measurement** |
| --- | --- | --- | --- | --- | --- | --- |
| Air-Polishing | Glycine | Huang. 2019 | **T**: 0.07±0.01  **C**: 0.07±0.00  **𝛿**: 0 (0%) | NA | Medium power. distance 0.5-1cm | Profilometer |
|  | Glycine | Tan. 2022 | NA | **T**:1.79  **C**:1.61  **𝛿**:0.18 (11%) | 1min. distance 5-10mm. SLA surface | Microscope |
| Curette | Carbon Reinforced Plastic | Huang. 2019 | **T**: 0.11±0.03  **C**: 0.07±0.00  **𝛿**: 0.04 (57%) | NA | Overlapping strokes | Profilometer |
|  | Plastic | Lang. 2016 | **T**: 0.93±0.048  **C**: 0.90±0.039  **𝛿**: 0.03 (3%) | NA | 100strokes | Profilometer |
|  | Plastic | Lang. 2016 | **T**: 0.93±0.082  **C**: 0.90±0.039  **𝛿**: 0.03 (3%) | NA | 20strokes | Profilometer |
|  | Plastic | Tan. 2022 | NA | **T**:1.81  **C**:1.61  **𝛿**:0.2 (12%) | Overlapping strokes. SLA surface | Microscope |
|  | Titanium | Huang. 2019 | **T**: 0.13±0.06*  **C**: 0.07±0.00  **𝛿**: 0.06 (86%) | NA | Overlapping strokes | Profilometer |
|  | Titanium | Lang. 2016 | **T**: 0.90±0.144  **C**: 0.90±0.039  **𝛿**: 0 (0%) | NA | 100strokes | Profilometer |
|  | Titanium | Lang. 2016 | **T**: 0.85±0.335  **C**: 0.90±0.039  **𝛿**: -0.05 (-6%) | NA | 20strokes | Profilometer |
|  | Titanium | Tan. 2022 | NA | **T**:1.85  **C**:1.61  **𝛿**:0.24 (15%) | Overlapping strokes. SLA surface | Microscope |
| Laser | Diode | Lang. 2016 | **T**: 0.86±0.054  **C**: 0.90±0.039  **𝛿**: -0.04 (-4%) | NA | 60s. 1.4W | Profilometer |
|  | CO2 | Stübinger. 2008 | NA | **T**:0.3054±0.1922 **C**:0.0256±0.0050 **𝛿**:0.2798 (1093%) | 10s. 6W. 10600nm. distance 0.5-1mm | Microscope |
|  | CO2 | Stübinger. 2008 | NA | **T**:0.7399±0.1320 **C**:0.0256±0.0050 **𝛿**:0.7143 (2790%) | 20s. 4.5W. 10600nm. distance 0.5-1mm | Microscope |
|  | CO2 | Stübinger. 2008 | NA | **T**:0.4464±0.4367 **C**:0.0256±0.0050 **𝛿**:0.4208 (1644%) | 30s. 4.5W. 10600nm. distance 0.5-1mm | Microscope |
|  | CO2 | Stübinger. 2008 | NA | **T**:0.4004±0.6665 **C**:0.0256±0.0050  **𝛿**:0.3748 (1464%) | 60s. 4W. 10600nm. distance 0.5-1mm | Microscope |
|  | CO2 | Stübinger. 2008 | NA | **T**:0.8959±0.4368** **C**:0.0256±0.0050  **𝛿**:0.8703 (3400%) | 60s. 4.5W. 10600nm. distance 0.5-1mm | Microscope |
| Ultrasonic | Carbon | Huang. 2019 | **T**: 0.08±0.01  **C**: 0.07±0.00  **𝛿**: 0.01 (14%) | NA | Overlapping strokes | Profilometer |
|  | Metal | Tan. 2022 | NA | **T** :2.05 *  **C** :1.61  **𝛿**: 0.44 (27%) | 1min. power 70%. SLA surface | Microscope |

T= test group. C= control group. 𝛿= difference between test and control group. NA: Not Available *= p< 0.05. **=p>0.01. SLA= sandblasted acid-etched

Supplementary Table 7. Surface roughness values on mixed surfaces (µm. mean ± SD)

| **Instrument** | **Type** | **Article** | **Ra** | **Sa** | **Rz** | **Protocol** | **Measurement** |
| --- | --- | --- | --- | --- | --- | --- | --- |
| Brush | Titanium | Lang. 2016 | **T**: 1.37±0.062 **  **C**: 1.03±0.077  **𝛿**: 0.34 (33%) | NA | NA | 60s | Profilometer |
| Curette | Plastic | Lang. 2016 | **T**: 1.11±0.037  **C**: 1.03±0.077  **𝛿**: 0.09 (9%) | NA | NA | 20strokes | Profilometer |
|  | Plastic | Lang. 2016 | **T**: 1.13±0.023  **C**: 1.03±0.077  **𝛿**: 0.1 (10%) | NA | NA | 100strokes | Profilometer |
|  | Titanium | Lang. 2016 | **T**: 1.42±0.174  **C**: 1.03±0.077  **𝛿**: 0.39 (38%) | NA | NA | 20strokes | Profilometer |
|  | Titanium | Lang. 2016 | **T**: 1.50±0.192  **C**: 1.03±0.077  **𝛿**: 0.47 (46%) | NA | NA | 100strokes | Profilometer |
| Laser | Diode | Lang. 2016 | **T**: 1.31±0.108**  **C**: 1.03±0.077  **𝛿**: 0.28 (27%) | NA | NA | 60s. 1.4W | Profilometer |

T= test group. C= control group. 𝛿= difference between test and control group. NA: Not Available *= p< 0.05. **=p>0.01
